# Supplementary material for: Community health worker intervention to reduce worker exposure to volatile organic compounds in small business auto and beauty shops in a marginalized community: A cluster randomized controlled trial
Source: PLoS One. 2026 Apr 27;21(4):e0346356. doi: 10.1371/journal.pone.0346356 (PMC13120284; doi:10.1371/journal.pone.0346356)
Supplement: S1 Checklist — (PDF) [file pone.0346356.s001.pdf]

CONSORT 2010 Checklist - Cluster Randomized Trial

Community health worker intervention to reduce worker exposure to volatile organic compounds in small business auto and beauty shops in a marginalized community: a cluster randomized controlled trial

Note: line numbers may be slightly off after formatting for submission, but they should be close.

Section/Topic	Item	Checklist Item & Extension	Page	Line	Notes
TITLE AND ABSTRACT					
Title	1a	Identification as a cluster randomized trial in the title	1	1-3	Complete. Title explicitly states "cluster randomized controlled trial".
Abstract	1b	Structured summary of trial design, methods, results, and conclusions following CONSORT for abstracts extension	2	15-36	Complete. Abstract includes: trial design (two-arm, parallel, cluster randomized trial with shops as clusters); shop eligibility (small business ≤25 employees, auto repair or beauty shops); CHW-led intervention (assessment, knowledge provision, $300 budget for controls); primary outcome (change in TVOCs at shop level); randomization (immediate vs delayed intervention, stratified by sector); blinding (not explicitly stated in abstract but addressed in Methods); number randomized (38 auto, 46 beauty shops); recruitment (dates provided in Results); results (non-significant intervention effects with specific point estimates and CIs); funding (mentioned in separate section at end); trial registration (NCT03455530).
INTRODUCTION					
Background	2a	Scientific background and rationale for using a cluster design	2-4	40-87; 111-113	Complete. Background section states small businesses face barriers to implementing workplace health protections; Latino workers disproportionately affected by occupational diseases; CHWs can overcome challenges through community trust; auto and beauty shops employ marginalized workers with VOC exposures. Methods (p. 4, lines 111-113) section explicitly states cluster

Section/Topic	Item	Checklist Item & Extension	Page	Line	Notes
					design ensured "logistical simplicity and consistency in intervention application across all workers within a shop and minimized contamination risks."
Objectives	2b	Specific objectives and whether they pertain to cluster level, individual level, or both	3	81-84	Complete. Specific objective stated at end of Background section: “To test whether such a CHW-led intervention could reduce total VOC exposure (primary outcome) and lower specific VOC hazard scores (secondary outcome) in small auto repair and beauty shops in low-income, predominantly Latino neighborhoods in the Tucson, AZ metropolitan area, we used a cluster randomized trial, in which clusters were shops.” Primary outcome (TVOC) analyzed at shop level (cluster), as stated explicitly in Abstract: "The primary outcome was the change in TVOCs at the shop level after the intervention."
METHODS					
Trial design	3a	Description of trial design (including allocation ratio) with definition of cluster and how design features apply to clusters	4-5	111-126; 163-165	Complete. Methods section describes: (1) two-arm, parallel, cluster randomized trial design; (2) clusters defined as shops; (3) 1:1 allocation to immediate vs delayed intervention groups (ethical rationale to allow all participants to benefit); (4) stratified randomization by shop subtype (Auto No Paint, Beauty Hair and Nails, Beauty Hair Only); (5) target of 3 assessments per shop with 4 workshift measurements per assessment.
Trial design	3b	Important changes to methods after trial commencement (such as eligibility criteria), with reasons	4	129-149	Complete. Description of COVID-19 pause in February 2020 and restart in January 2022. Methodological changes made: (1) removed direct UA researcher observation throughout workshifts due to pandemic hesitancy, replaced with self-reported activity logs with periodic UA team check-ins; (2) removed tube connected to

Section/Topic	Item	Checklist Item & Extension	Page	Line	Notes
					PID because it frequently kinked, causing data loss; (3) timeline for completing assessments extended from original 6 months to up to 21 months due to scheduling challenges, temporary closures, and delays in shops choosing controls; (4) geographic recruitment expanded from specific ZIP codes to similar socioeconomic areas within metro region due to recruitment difficulties.
Participants	4a	Eligibility criteria for clusters and participants	3	99-109	Complete. Cluster eligibility: small businesses (≤25 employees), auto repair or beauty shops, in specific Tucson AZ ZIP codes with high percentage Latino population (later expanded to similar socioeconomic areas). Participant eligibility: 18+ years old, able to speak/read/write Spanish or English, worked at participating shops. Exclusion criteria not explicitly stated but implied (workers under 18, non-Spanish/English speakers).
Participants	4b	Settings and locations where data were collected	3; 7	90-91; 147-149; 272-274	Complete. Setting: Tucson, AZ metropolitan area. Initially specific ZIP codes with high percentage Latino population, expanded to similar socioeconomic areas. Collaboration between SERI (community-based organization), El Rio Community Health Center, and University of Arizona. Data collection from January 2022 to January 2024.
Interventions	5	Interventions with sufficient detail for replication; whether they pertain to cluster, individual, or both levels	4	150-157	Complete. Cluster-level intervention delivered by CHWs: (1) assessed shop services and product inventories for VOC content; (2) reviewed and suggested VOC exposure controls with employees and owners; (3) provided up to $300 per shop for purchasing controls; (4) followed hierarchy of controls (elimination/substitution first, then engineering

Section/Topic	Item	Checklist Item & Extension	Page	Line	Notes
					controls like air purifiers, PPE last); (5) owners made final decisions on which controls to implement. Some controls implemented immediately (e.g., keeping containers closed), others required purchase.
Outcomes	6a	Pre-specified primary and secondary outcomes; whether they pertain to cluster, individual, or both levels	5	158-195	Complete. Primary outcome: change in TVOCs at shop level (cluster) assessed across 3 timepoints with up to 4 workshift measurements per assessment using photoionization detectors (ppbRAE 3000). TVOCs measured real-time every 20 seconds, aggregated to workshift time-weighted averages. Secondary outcome: hazard scores derived from specific VOC concentrations measured via Summa canisters, compared to occupational exposure limits. Both outcomes at shop level.
Outcomes	6b	Changes to trial outcomes after commencement (with reasons)	N/A	N/A	No changes to trial outcomes after commencement.
Sample size	7a	Sample size determination with cluster-specific parameters (ICC, cluster size, calculation method)		142-149	Mostly complete but with acknowledged limitations. Target sample size: 60 shops per sector (auto and beauty). Power calculation: 80% power to detect 47% change in VOC concentrations (exceeded assumed minimum important difference of 20%, based on 59-98% reductions seen in specific VOCs with alternative spray gun cleaner). Critical limitation openly stated: "Detailed calculation assumptions (including assumed intracluster correlation coefficient (ICC) values) are no longer available because of personnel changes." Observed ICCs reported: 0.32 for auto shops, 0.39 for beauty shops. Achieved sample: 38 auto shops, 46 beauty shops.
Sample size	7b	When applicable, explanation of any interim analyses and stopping guidelines	N/A	N/A	N/A

Section/Topic	Item	Checklist Item & Extension	Page	Line	Notes
Randomization: Sequence generation	8a	Method used to generate random allocation sequence	4	123-124	Complete. States "A UA statistician created a computer-generated randomization list for each subtype of shop".
Randomization: Sequence generation	8b	Type of randomization with details of stratification or matching if used	4	115; 123-126	Complete. Randomization type: stratified by shop subtype (Auto No Paint [ANP], Beauty Hair and Nails [BN], Beauty Hair Only [BH]). Allocation ratio: 1:1 to immediate or delayed intervention group.
Allocation concealment	9	Allocation concealment mechanism; specification that allocation was at cluster level	4	123-126	Complete, although no specific concealment mechanism. Randomization lists created by UA statistician and sent to CHWs at SERI at start of study. Allocation at cluster (shop) level.
Implementation	10a	Who generated random allocation sequence, who enrolled clusters, who assigned clusters to interventions	3-4	90-99; 123-126	Complete. Sequence generation: UA statistician. Cluster enrollment: SERI CHWs conducted recruitment. Cluster assignment: UA statistician created randomization lists and sent to CHWs at SERI; CHWs then enrolled shops according to the randomization scheme.
Implementation	10b	Mechanism by which individuals were included in clusters	3	99-109	Complete. Individual participants (workers) included in clusters (shops) based on: (1) shop participation; (2) individual eligibility (18+ years old, Spanish or English speaking/reading/writing); (3) individual informed consent given after shop randomization. Workers received incentives: $10 cash per workshift for PID use; personal VOC data, shop VOC data, free health screenings, resources for follow-up care and insurance navigation.
Implementation	10c	From whom consent was sought; whether before or after randomization	3	106-109	Complete. Consent sought from individual participants (workers) after shop randomization. IRB approval from UA Human Subjects Protection Program (#1709821542) documented.
Blinding	11a	If done, who was blinded after assignment to intervention and how	4	126-128	Complete but no blinding. Explicitly states: "Because

Section/Topic	Item	Checklist Item & Extension	Page	Line	Notes
					the intervention was often visible (e.g., a new air purifier), neither the participants nor the UA team could be guaranteed to remain blinded to the intervention status."
Blinding	11b	If relevant, description of the similarity of interventions	N/A	N/A	Not applicable. This item addresses similarity of interventions in blinding scenarios. Given that study used delayed control design (not placebo or sham) and explicitly acknowledged inability to maintain blinding, similarity of interventions is not relevant. Delayed control group would eventually receive same intervention as immediate group.
Statistical methods	12a	Statistical methods used to compare groups for primary and secondary outcomes; how clustering was taken into account	6-7	229-269	Complete. Primary outcome (TVOCs): Linear mixed-effects models with log-transformed TVOC as outcome; fixed effects for intervention group (immediate/delayed), assessment time (1,2,3), and their interaction; random effects for shop (intercept) and assessment time within shop (intercept) to account for correlations within shop and stronger correlations within shop at same assessment. Separate models for auto and beauty shops. Both unadjusted and adjusted models fitted. Contrasts tested change from pre- to post-intervention between arms. Secondary outcome (hazard scores): similar mixed models but without random effect for workshift within assessment. Statistical software: R 4.4.1 with lme4 package.
Statistical methods	12b	Methods for additional analyses, such as subgroup analyses and adjusted analyses	6-7	242-266	Complete. Covariate selection process described: (1) logical relevance to outcome and potential impact on exposures, and (2) baseline imbalances between groups. For auto shops, adjusted model included baseline outside ventilation indicator and average workshift apparent temperature. For beauty

Section/Topic	Item	Checklist Item & Extension	Page	Line	Notes
					shops, adjusted model included hair-and-nail shop indicator and baseline air exchange rate (ACH). Rationale for each covariate provided in text and supplemental materials. Alternate adjusted models also explored (mainly in Supplemental Material) with covariates that changed the treatment effect by more than 10% when added one-at-a-time to unadjusted model. Analysis of specific VOC hazard scores using similar mixed model approach.
RESULTS					
Participant flow	13a	For each group, the numbers of clusters randomly assigned, received intended treatment, and analyzed for the primary outcome	8; 12	287-289; 327	Complete. Figure 1 (CONSORT flow diagram) shows: 38 auto shops and 46 beauty shops enrolled and randomized. Allocation: 19 auto shops to each arm; 23 beauty shops to each arm. All randomized shops received intended treatment (intervention delivered as assigned), unless they dropped out before intervention was given (as shown in diagram). Analysis: 846 workshift measurements at 236 shop assessments. Analysis by intention-to-treat at shop level.
Participant flow	13b	For each group, losses and exclusions for clusters and individuals	6-7	277-289	Complete. Cluster losses: 6/84 (7%) shops dropped out with detailed reasons: (1) owner no longer interested; (2) shop under renovation; (3) shop sold and new owner declined; (4) shop changed from auto to restaurant; (5) shop closed. Distribution: 4 auto shops, 2 beauty shops. Timing: 1 auto shop before first assessment, 2 auto shops between assessments 1-2, 1 auto shop between assessments 2-3, 2 beauty shops between assessments 1-2. Additional exclusions: 3 shops (4%) moved during study; data after move excluded from analysis but retained for descriptive purposes (BH021, BH009,

Section/Topic	Item	Checklist Item & Extension	Page	Line	Notes
					BH028). Individual participant losses not separately quantified beyond shop-level reporting – the study was not tracking individuals but rather shifts at shops with outcomes at shop level.
Recruitment	14a	Dates defining periods of recruitment and follow-up	7	272-274	Complete. Recruitment period: CHWs recruited from January 10, 2022 to May 11, 2023 (shops). Data collection period: UA team collected TVOC, specific VOC, and supplemental data from January 15, 2022 to January 18, 2024.
Recruitment	14b	Why trial ended or was stopped	7	274-276	Complete. Trial stopped before reaching target sample size (60 shops per sector) for two reasons: (1) COVID-19 pandemic caused delays (2) increased difficulty recruiting businesses in post-pandemic period. Administrative/logistical decision rather than efficacy or safety concerns.
Baseline data	15	Baseline characteristics for individual and cluster levels	8-11	291-315	Complete. Table 1 presents baseline cluster (shop) characteristics for auto and beauty shops that participated, by sector and intervention group. Table 2 presents baseline demographics for auto and beauty shop workers who used a PID monitor to measure TVOCs, by sector and intervention group.
Numbers analyzed	16	For each group, number of clusters included in each analysis	7; 12	277-289; 327-328	Complete. 846 workshift measurements at 236 shop assessments from 83 shops (total of 84 shops, but 1 shop dropped out before any assessments) included in analysis. Breakdown by sector: 376 auto workshifts at 106 auto assessments; 470 beauty workshifts at 130 beauty assessments. Separate analyses conducted for auto and beauty sectors. Analysis dataset accounts for 6 shop dropouts and 3 shop exclusions (moved), but all randomized shops included in intention-to-treat analysis

Section/Topic	Item	Checklist Item & Extension	Page	Line	Notes
					up to point of dropout/exclusion.
Outcomes and estimation	17a	Results at appropriate level with estimated effect size, precision (e.g., 95% CI), and ICC for each primary outcome	12-13	343-359	Complete. Table 3 reports intervention effect sizes and 95% CIs for both auto and beauty shops. Neither effect statistically significant. ICCs reported: 0.32 for auto shops, 0.39 for beauty shops. Variance components detailed in Supplemental Table S4.
Outcomes and estimation	17b	For binary outcomes, both absolute and relative effect sizes	N/A	N/A	Not applicable. Primary and secondary outcomes are continuous (TVOC concentration and hazard score derived from specific VOC concentrations), not binary. No binary outcomes analyzed in this trial.
Ancillary analyses	18	Results of other analyses including subgroup and adjusted analyses	13-15	348-430	Complete. Additional analyses reported: (1) Unadjusted models presented alongside adjusted models; (2) Alternate adjusted models explored with different covariates (Supplemental Materials); (3) Comparison between sectors: beauty shops had TVOC concentrations approximately 10 times higher than auto shops; (4) Subgroup analysis within beauty shops: hair-and-nail shops had ~3 times higher TVOCs than hair-only shops; (5) Analysis of hazard scores derived from specific VOCs as secondary outcome. (6) Ventilation analysis: 87% of beauty shop assessments had air exchange rates below recommended minimum of 4 ACH.
Harms	19	Important harms or unintended effects in each group	N/A	N/A	Not explicitly reported in paper. Given intervention nature (CHW education + low-cost equipment like air purifiers), serious harms unlikely. No adverse events reported.
DISCUSSION					
Limitations	20	Trial limitations addressing sources of potential bias and imprecision	17-18	528-542	Complete. Limitations discussion: (1) Recruited less than targeted number of shops, which reduced power; (2) PIDs did not

Section/Topic	Item	Checklist Item & Extension	Page	Line	Notes
					sample TVOCs directly in breathing zone; (3) Hazard scores underestimated exposure risk. (4) Bias could arise from UA site audit (team could infer controls); (5) Bias could arise from regular UA team visits during workshift (participants reminded to reduce VOCs by e.g., turning on fan).
Generalizability	21	Generalizability to clusters and/or individuals (as relevant)	15-18	431-554	Complete. CHW model demonstrated 93% retention rate, suggesting approach is acceptable and scalable to similar small businesses. Intervention cost ($300 per shop) is feasible. Study results specific to small auto/beauty shops in Latino communities in Southwestern US, but results may generalize to similar marginalized worker populations in under-resourced small businesses. High VOC variability likely common in auto and beauty shops.
Interpretation	22	Interpretation consistent with results, balancing benefits and harms, and considering other relevant evidence	15-18	431-554	Interpretation acknowledges non-significant results but discusses practical significance. For beauty shops, confidence interval includes clinically meaningful 20% reduction considered minimum important difference. Auto shop results more uncertain with wider confidence intervals. Emphasizes genuine contributions: (1) documentation of exposure disparities (beauty 10x higher than auto); (2) inadequate ventilation in 87% of beauty shop assessments; (3) feasibility of CHW-led approach with 93% retention; (4) rare high-quality VOC exposure data in understudied occupational settings. Discusses mechanisms for lack of statistical significance (variability, power, implementation challenges). Balances benefits (knowledge generation, feasibility demonstration, exposure characterization) against

Section/Topic	Item	Checklist Item & Extension	Page	Line	Notes
					lack of definitive intervention effect. No harms to balance.
OTHER INFORMATION					
Registration	23	Registration number and name of trial registry	2	36	Complete. Trial registration: ClinicalTrials.gov ID NCT03455530, registered March 6, 2018. Registration clearly stated in Abstract. Registration occurred before trial start (February 2020 original start; January 2022 restart after COVID pause).
Protocol	24	Where full trial protocol can be accessed, if available	3-7; S2_Protocol.pdf	88-268	Complete. A copy of the protocol that was approved by the ethics committee has been submitted as supplementary information file S2_Protocol_IRB.pdf. Additionally, a summary of the protocol and substantive modifications (e.g., COVID-19 pause, methodological changes, timeline extensions, and geographic expansion to it) documented in Methods.
Funding	25	Sources of funding and role of funders	19	597-600	Complete. Funding sources: NIH/NIEHS R01 ES028250 (primary), P30 ES006694 (Southwest Environmental Health Sciences Center), T32 ES007091 (training grant), R25 ES025060.
