# Supplementary material for: Community health worker intervention to reduce worker exposure to volatile organic compounds in small business auto and beauty shops in a marginalized community: A cluster randomized controlled trial
Source: PLoS One. 2026 Apr 27;21(4):e0346356. doi: 10.1371/journal.pone.0346356 (PMC13120284; doi:10.1371/journal.pone.0346356)
Supplement: S2 File — (PDF) [file pone.0346356.s002.pdf]

Supplemental Material

Controls (VOC reduction strategies)

Table SM1 presents data from SERI on the controls shops agreed to or were already doing, by sector and intervention group.

Table SM1: Controls for auto and beauty shops that participated, by sector and intervention group.

	Auto				Beauty			
	Intervention group				Intervention group			
	**Immediate** (N = 19)[1]	**Delayed** (N = 19)[1]	**Total** (N = 38)[1]	**p-value**	**Immediate** (N = 23)[1]	**Delayed** (N = 23)[1]	**Total** (N = 46)[1]	**p-value**
**General controls agreed to**								
**Keep containers closed when not in use**				0.7[2]				>0.9[2]
Yes	2 (11%)	3 (18%)	5 (14%)		11 (48%)	10 (43%)	21 (46%)	
No	0 (0%)	0 (0%)	0 (0%)		0 (0%)	0 (0%)	0 (0%)	
Already Doing	16 (89%)	14 (82%)	30 (86%)		12 (52%)	13 (57%)	25 (54%)	
Unknown	1	2	3					
**Purchase smaller containers to minimize waste**				**0.015**[2]				0.5[2]
Yes	2 (11%)	9 (53%)	11 (31%)		10 (43%)	6 (26%)	16 (35%)	
No	4 (22%)	4 (24%)	8 (23%)		5 (22%)	6 (26%)	11 (24%)	
Already Doing	12 (67%)	4 (24%)	16 (46%)		8 (35%)	11 (48%)	19 (41%)	
Unknown	1	2	3					
**Dispose of hazardous waste properly**				0.6[2]				>0.9[2]
Yes	1 (5.6%)	2 (12%)	3 (8.6%)		9 (39%)	9 (39%)	18 (39%)	
No	0 (0%)	0 (0%)	0 (0%)		0 (0%)	0 (0%)	0 (0%)	
Already Doing	17 (94%)	15 (88%)	32 (91%)		14 (61%)	14 (61%)	28 (61%)	
Unknown	1	2	3					
**Place trash soaked with chemicals in trash can with tight lid**								0.8[2]
Yes					13 (57%)	15 (65%)	28 (61%)	
No					0 (0%)	0 (0%)	0 (0%)	
Already Doing					10 (43%)	8 (35%)	18 (39%)	
Unknown								
**Maintain drum lids tight-fitting, sealed, and bungs closed**				0.2[2]				
Yes	2 (12%)	6 (35%)	8 (24%)					
No	0 (0%)	0 (0%)	0 (0%)					
Already Doing	15 (88%)	11 (65%)	26 (76%)					
Unknown	2	2	4					
**Store rags soaked with chemicals in sealed containers**				0.2[2]				
Yes	2 (11%)	5 (29%)	7 (20%)					
No	2 (11%)	0 (0%)	2 (5.7%)					
Already Doing	14 (78%)	12 (71%)	26 (74%)					
Unknown	1	2	3					
**Keep lids on solvent parts washers closed when not in use**				**0.008**[2]				
Yes	2 (11%)	9 (60%)	11 (33%)					
No	1 (5.6%)	0 (0%)	1 (3.0%)					
Already Doing	15 (83%)	6 (40%)	21 (64%)					
Unknown	1	4	5					
**Use the sample provided**	9 (75%)	4 (57%)	13 (68%)	0.6[2]	21 (95%)	21 (100%)	42 (98%)	>0.9[2]
Unknown	7	12	19		1	2	3	
**Take a training class**	0 (0%)	0 (0%)	0 (0%)	>0.9[2]	0 (0%)	0 (0%)	0 (0%)	>0.9[2]
Unknown	1	2	3					
**Develop a new policy**[3]	1 (5.6%)	0 (0%)	1 (2.9%)	>0.9[2]	2 (8.7%)	4 (17%)	6 (13%)	0.7[2]
Unknown	1	2	3					

Table SM1: Controls for auto and beauty shops that participated, by sector and intervention group.

	Auto				Beauty			
	Intervention group				Intervention group			
	Immediate (N = 19)[1]	Delayed (N = 19)[1]	Total (N = 38)[1]	p-value	Immediate (N = 23)[1]	Delayed (N = 23)[1]	Total (N = 46)[1]	p-value
**Eliminate any procedures**[4]	1 (5.6%)	0 (0%)	1 (2.9%)	>0.9[2]	0 (0%)	1 (4.3%)	1 (2.2%)	>0.9[2]
Unknown	1	2	3					
**Eliminate any products**[5]	1 (5.6%)	0 (0%)	1 (2.9%)	>0.9[2]	2 (8.7%)	5 (22%)	7 (15%)	0.4[2]
Unknown	1	2	3					
**Ventilation controls and type(s) agreed to increase**								
**Increase natural or existing ventilation**	14 (78%)	16 (94%)	30 (86%)	0.3[2]	13 (57%)	19 (83%)	32 (70%)	0.11[2]
Unknown	1	2	3					
**Air purifier(s)**	0 (0%)	0 (0%)	0 (0%)	>0.9[2]	0 (0%)	2 (8.7%)	2 (4.3%)	0.5[2]
Unknown	1	2	3					
**Ceiling fan(s)**	1 (5.6%)	0 (0%)	1 (2.9%)	>0.9[2]	3 (13%)	4 (17%)	7 (15%)	>0.9[2]
Unknown	1	2	3					
**Exhaust system(s)**	0 (0%)	0 (0%)	0 (0%)	>0.9[2]	1 (4.3%)	0 (0%)	1 (2.2%)	>0.9[2]
Unknown	1	2	3					
**Open door(s)**	10 (56%)	12 (71%)	22 (63%)	0.5[2]	9 (39%)	10 (43%)	19 (41%)	>0.9[2]
Unknown	1	2	3					
**Open window(s)**	7 (39%)	10 (59%)	17 (49%)	0.3[2]	8 (35%)	14 (61%)	22 (48%)	0.14[2]
Unknown	1	2	3					
**Portable fan(s)**	11 (61%)	10 (59%)	21 (60%)	>0.9[2]	2 (8.7%)	3 (13%)	5 (11%)	>0.9[2]
Unknown	1	2	3					
**Additional PPE controls and type(s) agreed to increase**								
**Use additional PPE**	16 (89%)	16 (94%)	32 (91%)	>0.9[2]	15 (65%)	19 (83%)	34 (74%)	0.3[2]
Unknown	1	2	3					
**Aprons**	1 (5.6%)	0 (0%)	1 (2.9%)	>0.9[2]	14 (61%)	14 (61%)	28 (61%)	>0.9[2]
Unknown	1	2	3					
**Glasses**	4 (22%)	6 (35%)	10 (29%)	0.5[2]	3 (13%)	5 (22%)	8 (17%)	0.7[2]
Unknown	1	2	3					
**Gloves**	16 (89%)	15 (88%)	31 (89%)	>0.9[2]	14 (61%)	13 (57%)	27 (59%)	>0.9[2]
Unknown	1	2	3					
**Masks**	4 (22%)	9 (53%)	13 (37%)	0.086[2]	10 (43%)	16 (70%)	26 (57%)	0.14[2]
Unknown	1	2	3					
**Program-purchased controls**								
**Total cost of program-purchased controls ($)**				0.2[6]				>0.9[6]
Mean (SD)	276 (22)	256 (52)	267 (40)		284 (24)	284 (21)	284 (22)	
Min - Max	229 - 300	167 - 343	167 - 343		226 - 323	240 - 323	226 - 323	
Unknown	1	2	3					
**Engineering control - air purifier**	2 (11%)	2 (11%)	4 (11%)	>0.9[2]	20 (87%)	20 (87%)	40 (87%)	>0.9[2]
**Engineering control - exhaust system**	0 (0%)	0 (0%)	0 (0%)	>0.9[2]	0 (0%)	1 (4.3%)	1 (2.2%)	>0.9[2]
**Engineering control - fan**	12 (63%)	9 (47%)	21 (55%)	0.5[2]	1 (4.3%)	1 (4.3%)	2 (4.3%)	>0.9[2]
**Engineering control - new equipment**[7]	8 (42%)	13 (68%)	21 (55%)	0.2[2]	0 (0%)	1 (4.3%)	1 (2.2%)	>0.9[2]
**Engineering control - trash can with lid**	0 (0%)	0 (0%)	0 (0%)	>0.9[2]	15 (65%)	15 (65%)	30 (65%)	>0.9[2]
**PPE**	14 (74%)	9 (47%)	23 (61%)	0.2[2]	14 (61%)	16 (70%)	30 (65%)	0.8[2]
**Product substitution**	0 (0%)	0 (0%)	0 (0%)	>0.9[2]	2 (8.7%)	2 (8.7%)	4 (8.7%)	>0.9[2]

P-values < 0.05 appear in bold.

[1] n (%) for categorical variables, mean (SD) and range for continuous variables; n for unknown. SD: standard deviation.

[2] Fisher's Exact Test for Count Data

[3] One auto shop agreed to a new policy to not use solvents - they will use soapy water in a hot tank instead. Four beauty shops that agreed to a new policy stated they would limit Brazilian blowouts; one beauty shop stated, 'They will try to reduce activities that they use from their inventory'; one beauty shop did not state their policy.

[4] The auto shop that agreed to eliminate a procedure did not state what it was. One beauty shop agreed to eliminate the Brazilian blowout procedure.

[5] One auto shop agreed to eliminate brake cleaner, carburetor cleaner, and solvents. For beauty shops, one agreed to eliminate Aquage taffy, Kenra frenzy, and Kenra paste; one agreed to eliminate Bedhead hair spray; one agreed to try organic products; one agreed to eliminate Kenra; one agreed to eliminate Shine N Jam; one agreed to eliminate sprays; and one agreed to substitute high-VOC sprays with low-VOC sprays using recommendations from the Environmental Working Group (EWG).

[6] Welch Two Sample t-test

[7] New equipment included things like refillable spray bottles, non-aerosol sprayers, and hydrophobic mop heads, which should reduce VOCs in the shop air.

Primary outcome: TVOCs

Plots of data: average shop TVOCs at each assessment

Figure SM1 shows violin plots of average TVOCs at each shop at each assessment for each intervention group.

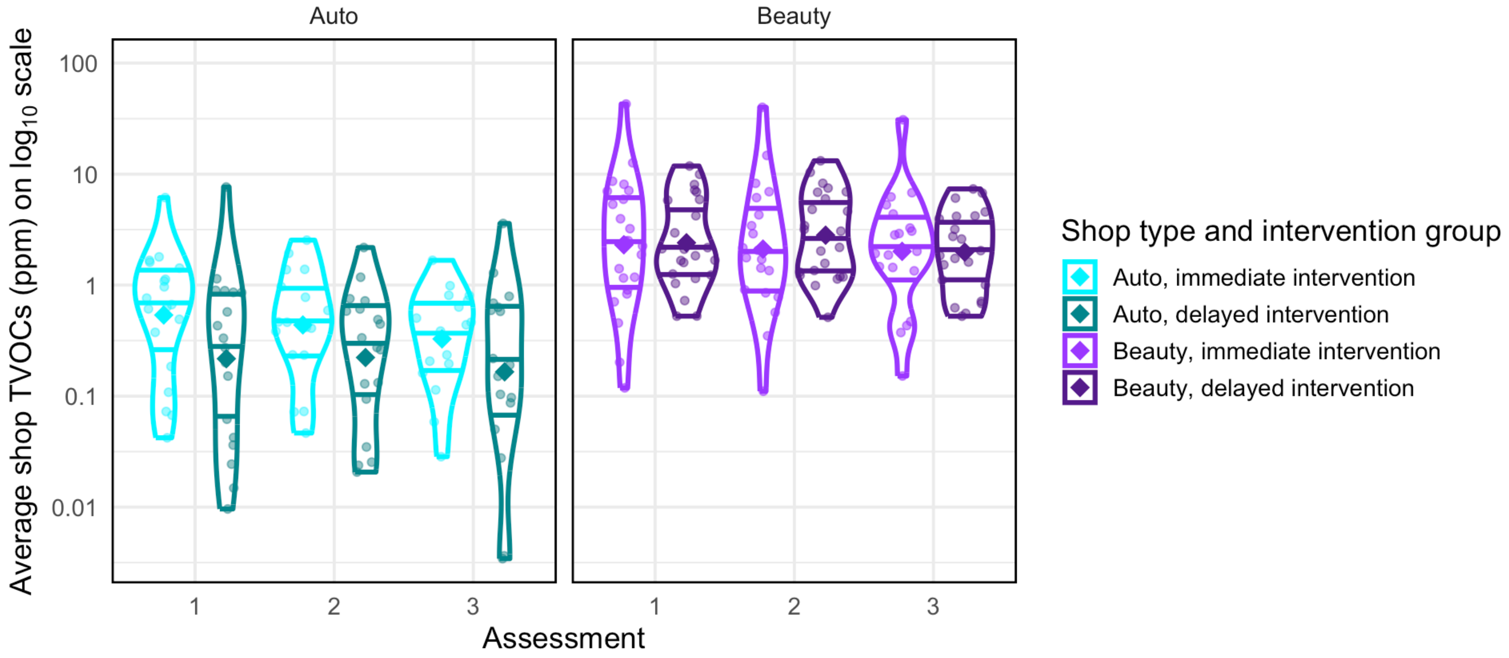

Figure SM1: Each datapoint in this plot represents average TVOCs data at one shop at each assessment: it is the geometric mean of the TWA TVOCs for each shift of that shop for that assessment. Horizontal lines in the violin plots represent the 25th percentile (Q1), 50th percentile (median), and 75th percentile (Q3). The diamond represents the geometric mean. The shape of the violin represents the relative frequency of data points at different values, based on a smoothed kernel density estimation, and the bounds correspond to the range over which the density is estimated (typically the range of the data).

Mixed models for TVOCs

Specifications for each model are given Table SM2. The “Auto Adjusted” and “Beauty Adjusted” models are the adjusted models with results presented in the main body of the paper. The “Variant” models are the alternate models mentioned in the paper with covariates chosen because they changed the treatment effect by 10% or more when included one-at-a-time in the unadjusted model. Table SM3 presents estimated geometric means (GMs) of TVOCs and their 95% CIs along with estimated intervention effects for each model, and Figure SM2 plots the estimated GM TVOCs with their 95% CIs for each model. Table SM4 presents measures of the variation for each model.

Table SM2: TVOCs mixed model specifications for auto and beauty shops. Each model is of log-transformed concentration of TVOCs on the fixed effects and random effects listed for that model. Values beyond baseline of possible post-treatment outcome variables (e.g., outside ventilation, air exchange rate) were not considered for inclusion in adjusted models.

Model	Reason	Fixed effects	Random effects
Auto Unadjusted		Intervention group<br>Assessment<br>Intervention group*Assessment	Shop<br>Assessment within shop
Auto Adjusted	Added all covariates that we expected might matter	Intervention group<br>Assessment<br>Intervention group*Assessment<br>Shop-level baseline outside ventilation<br>Shift-level apparent temperature	Shop<br>Assessment within shop
Auto Adjusted (Variant)	Added all covariates that when included one-at-a-time in unadjusted model changed the treatment effect by 10% or more	Intervention group<br>Assessment<br>Intervention group*Assessment<br>Shop-level baseline outside ventilation<br>Shop-level self-report administration activity<br>Shift-level apparent temperature	Shop<br>Assessment within shop
Beauty Unadjusted		Intervention group<br>Assessment<br>Intervention group*Assessment	Shop<br>Assessment within shop
Beauty Adjusted	Added all covariates that we expected might matter	Intervention group<br>Assessment<br>Intervention group*Assessment<br>Shop-level log-transformed baseline air exchange rate<br>Shop-level beauty shop type (hair and nails vs. hair only)	Shop<br>Assessment within shop
Beauty Adjusted (Variant)	Added all covariates that when included one-at-a-time in unadjusted model changed the treatment effect by 10% or more	Intervention group<br>Assessment<br>Intervention group*Assessment<br>Shop-level self-report administration activity<br>Shop-level self-report hair-processing activity	Shop<br>Assessment within shop

Table SM3: Estimated geometric means of TVOCs and their 95% CIs for each combination of intervention group and assessment and for all intervention groups and all assessments, along with estimated intervention effects, from the mixed models for auto and beauty shops. Results have been back-transformed to the original scale from the log scale, which results in asymmetrical CIs and differences on the log scale have become ratios on the original scale. Three values of the estimated intervention effect are presented for each model. (1) The estimated intervention effect from assessments 1 & 2: (Immediate Time 2/Immediate Time 1) / (Delayed Time 2/Delayed Time 1). (2) The estimated intervention effect from assessments 2 & 3: (Delayed Time 3/Delayed Time 2) / (Immediate Time 3/Immediate Time 2). (3) The geometric mean of the prior two intervention effect estimates; this is the best estimate of the intervention effect for each model and what is presented in the main body of the paper. The estimated intervention effect should be evaluated by comparing to 1: a value less than 1 indicates that the intervention was effective in reducing TVOCs, a value greater than 1 indicates that the intervention may have increased TVOCs, and a 95% CI that includes 1 indicates that the effect of intervention was not statistically significant (so we cannot determine if it had a beneficial effect or not). Est. = Estimated; GM = geometric mean; TVOCs = total volatile organic compounds; ppb = parts per billion; CI: Confidence interval

Model	Intervention group	Assessment	Est. GM TVOCs in ppb (95% CI)	Est. intervention effect from assessments 1 & 2 (95% CI)	Est. intervention effect from assessments 2 & 3 (95% CI)	Est. GM intervention effect from all assessments (95% CI)
Auto Unadjusted	Immediate	1	510 (260, 1001)	0.78 (0.28, 2.18)	0.96 (0.33, 2.75)	0.86 (0.35, 2.13)
	Delayed	1	201 (100, 405)			
	Immediate	2	436 (215, 886)			
	Delayed	2	221 (110, 443)			
	Immediate	3	314 (153, 646)			
	Delayed	3	152 (75, 310)			
	All	All	279 (187, 417)			
Auto Adjusted	Immediate	1	296 (129, 680)	1.16 (0.44, 3.09)	1.42 (0.52, 3.89)	1.28 (0.54, 3.03)
	Delayed	1	175 (87, 353)			
	Immediate	2	362 (155, 844)			
	Delayed	2	185 (92, 371)			
	Immediate	3	206 (84, 501)			
	Delayed	3	149 (73, 304)			
	All	All	218 (131, 362)			
Auto Adjusted (Variant)	Immediate	1	281 (122, 644)	0.95 (0.36, 2.50)	1.07 (0.39, 2.93)	1.01 (0.43, 2.38)
	Delayed	1	166 (83, 332)			
	Immediate	2	294 (124, 696)			
	Delayed	2	182 (91, 363)			
	Immediate	3	186 (77, 453)			
	Delayed	3	123 (59, 254)			
	All	All	196 (117, 329)			
Beauty Unadjusted	Immediate	1	2375 (1504, 3751)	0.76 (0.44, 1.33)	0.72 (0.40, 1.27)	0.74 (0.46, 1.20)
	Delayed	1	2422 (1534, 3824)			
	Immediate	2	2112 (1307, 3412)			
	Delayed	2	2817 (1783, 4451)			
	Immediate	3	2154 (1325, 3499)			
	Delayed	3	2057 (1288, 3285)			
	All	All	2309 (1726, 3089)			
Beauty Adjusted	Immediate	1	3081 (1983, 4786)	0.76 (0.44, 1.32)	0.70 (0.40, 1.24)	0.73 (0.45, 1.19)
	Delayed	1	3330 (2125, 5219)			
	Immediate	2	2738 (1720, 4358)			
	Delayed	2	3875 (2471, 6077)			
	Immediate	3	2821 (1758, 4527)			
	Delayed	3	2808 (1772, 4449)			
	All	All	3085 (2277, 4179)			
Beauty Adjusted (Variant)	Immediate	1	1695 (995, 2887)	0.88 (0.49, 1.60)	0.87 (0.48, 1.57)	0.88 (0.52, 1.47)
	Delayed	1	1799 (1064, 3044)			
	Immediate	2	1837 (1093, 3086)			
	Delayed	2	2204 (1334, 3643)			
	Immediate	3	1716 (990, 2975)			
	Delayed	3	1790 (1095, 2925)			
	All	All	1833 (1272, 2641)			

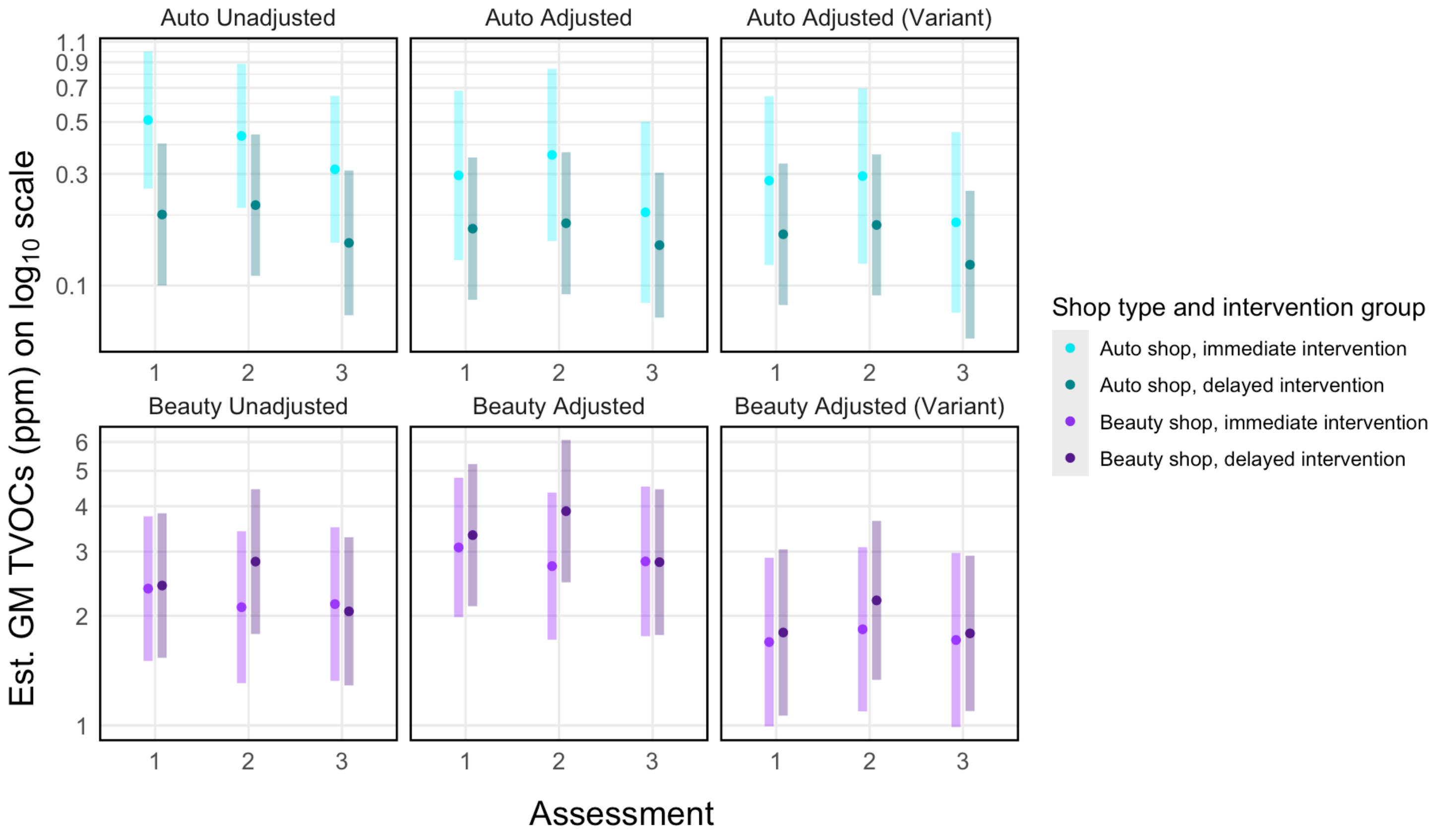

Figure SM2: Estimated geometric means of TVOCs and their 95% CIs for each mixed model for auto and beauty shops. Note that these means have been plotted on the $\log_{10}$ scale.

Table SM4: Variance components for the TVOC mixed models for auto and beauty shops. The "Percent total variance" for the Shop row represents the shop-level ICC, which quantifies the proportion of total variance attributable to differences between shops.

Model	Groups	Variance	Standard deviation	Percent total variance
Auto Unadjusted	Assessment within shop	0.63	0.79	18
	Shop	1.01	1.00	28
	Residual	1.93	1.39	54
Auto Adjusted	Assessment within shop	0.44	0.66	13
	Shop	1.13	1.06	32
	Residual	1.92	1.39	55
Auto Adjusted (Variant)	Assessment within shop	0.38	0.61	11
	Shop	1.16	1.08	33
	Residual	1.92	1.39	56
Beauty Unadjusted	Assessment within shop	0.22	0.47	13
	Shop	0.80	0.89	47
	Residual	0.70	0.83	41
Beauty Adjusted	Assessment within shop	0.22	0.47	15
	Shop	0.59	0.77	39
	Residual	0.70	0.83	46
Beauty Adjusted (Variant)	Assessment within shop	0.21	0.46	13
	Shop	0.76	0.87	46
	Residual	0.70	0.83	42

Secondary outcome: hazard scores derived from specific VOCs

Table of specific VOCs with their reference values and health effects

Table SM5 presents the following for each specific VOC measured by the Summa canisters using the TO-15 method: its reference value used for calculating hazard scores, the source of that reference value, and its short-term and long-term health effects.

Table SM5: Reference values and their sources along with short-term and long-term health effects for specific VOCs measured by Summa canisters using the TO-15 method. CAS # is the unique chemical identification number assigned by the Chemical Abstracts Service. Each reference value (Ref. value) was obtained from the US Environmental Protection Agency's Integrated Risk Information System. The primary value was the Inhalation Reference Concentration (RfC). For chemicals that did not have an RfC value, the following were used in the order: Reference Dose (RfD), Cancer Inhalation Unit Risk (IUR), and Cancer Oral Slope (OS). To convert oral dose to inhalation dose, 70 kg bodyweight and 20 m^3/day respiratory rate were assumed. A risk of 10^{-4} was assumed in using the cancer slope factors.

Chemical name	CAS #	Ref. value ($\mu g/m^3$)	Source of ref. value	Short-term health effects	Long-term health effects
1,1,1-Trichloroethane	71-55-6	5,000	RfC	Poor coordination, irregular heartbeat, low blood pressure, headache, eye and nose irritation	Drowsiness, confusion, slow heartbeat, liver damage
1,1,2,2-Tetrachloroethane	79-34-5	70.0	RfD	Headache, drowsiness, nausea	Liver damage, necrosis. 2B, possible carcinogen
1,1,2-Trichloroethane	79-00-5	6.25	IUR	Irritation of skin, eyes, upper respiratory tract, and stomach	Central nervous system impairment, liver damage, human carcinogen
1,1-Dichloroethane	75-34-3			Eye and upper respiratory tract irritation, irregular heartbeat, intoxication, dizziness, nausea, vomiting, salivation, sneezing, coughing	Liver and kidney damage
1,1-Dichloroethene	75-35-4	200	RfC	Eye and upper respiratory tract irritation, irregular heartbeat, intoxication, dizziness, nausea, vomiting, salivation, sneezing, coughing	Liver and kidney damage
1,2,4-Trichlorobenzene	120-82-1	35.0	RfD	Eye, skin, and upper respiratory tract irritation, dizziness	Liver and kidney damage
1,2,4-Trimethylbenzene	95-63-6	60.0	RfC	Eye, respiratory system and skin irritation, central nervous system depressant, headache, fatigue, drowsiness	Bronchitis
1,2-Dibromoethane	106-93-4	9.00	RfD	Affects the skin	Affects liver, urinary system or kidneys, reproductive system. 2A, probable carcinogen
1,2-Dichlorobenzene	95-50-1	315	RfD	Upper respiratory and eye irritation	Liver damage
1,2-Dichloroethane	107-06-2	3.85	IUR	Nausea	Liver and kidney damage. 2B, possible carcinogen
1,2-Dichloropropane	78-87-5	4.00	RfC	Upper respiratory tract irritation, weight effects	Cardiac failure, death, carcinogen
1,3,5-Trimethylbenzene	108-67-8	60.0	RfC	Irritation of nose, throat, skin and eyes	Trouble breathing, cough, weakened nervous system, problems with blood
1,3-Butadiene	106-99-0	2.00	RfC	Affects skin and eyes	Carcinogen, neurological problems
1,3-Dichlorobenzene	541-73-1			Eye, skin, and respiratory irritation, headache, nausea, vomiting and diarrhea, vertigo, malaise, and unconsciousness	Liver and kidney damage. Reasonably anticipated to be a human carcinogen
1,4-Dichlorobenzene	106-46-7	800	RfC	Eye and skin irritation	Affects development, the liver, and urinary system. 2B, possible carcinogen
1,4-Dioxane	123-91-1	30.0	RfC	Eye and nose irritation	Liver and kidney damage and even death. 2B, possible carcinogen
2,2,4-Trimethylpentane	540-84-1			Transient central nervous system depression, respiratory depression, asphyxia, dizziness, headache, nausea, vomiting, dry skin, eye redness	May cause effects on the kidneys, liver and nervous system. If swallowed, aspiration into the lungs may result in chemical pneumonitis
2-Butanone (MEK)	78-93-3	5,000	RfC	Upper respiratory tract irritation, numbness and pain in hands and feet, eye and nasal irritation, headache, dizziness, vomiting	Central nervous system and peripheral nervous system impairment, neurotoxic effects
2-Hexanone	591-78-6	30.0	RfC	Eye and nose irritation	Affects the nervous system
2-Propanol (IPA)	67-63-0			Drowsiness, confusion, slow heartbeat, affects the liver and kidneys, sleepiness, poor	Brain damage

Table SM5: Reference values and their sources along with short-term and long-term health effects for specific VOCs measured by Summa canisters using the TO-15 method. CAS # is the unique chemical identification number assigned by the Chemical Abstracts Service. Each reference value (Ref. value) was obtained from the US Environmental Protection Agency's Integrated Risk Information System. The primary value was the Inhalation Reference Concentration (RfC). For chemicals that did not have an RfC value, the following were used in the order: Reference Dose (RfD), Cancer Inhalation Unit Risk (IUR), and Cancer Oral Slope (OS). To convert oral dose to inhalation dose, 70 kg bodyweight and 20 m^3/day respiratory rate were assumed. A risk of 10^{-4} was assumed in using the cancer slope factors.

**Chemical name**	**CAS #**	**Ref. value ($\mu g/m^3$)**	**Source of ref. value**	**Short-term health effects**	**Long-term health effects**
				coordination, skin and eye irritation, stomach pain, stomach bleeding, throwing up, cold skin, fast heartbeat	
4-Ethyltoluene	622-96-8			Drowsiness and dizziness	Unknown
4-Methyl-2-pentanone (MIK)	108-10-1	3,000	RfC	Irritation of nose and throat, dizziness and headache	Cancer
Acetone	67-64-1	3,150	RfD	Upper respiratory tract, eye, nose, throat, and lung irritation, dizziness, nausea, uncoordinated movement, slurred speech	Central nervous system impairment
Allyl chloride	107-05-1	1.00	RfC	Eye and upper respiratory tract irritation	Liver and kidney damage
Benzene	71-43-2	30.0	RfC	Drowsiness, dizziness, sleepiness, rapid heart rate, headache, tremors, confusion, unconsciousness, vomiting, stomach irritation	Leukemia, cancer
Benzyl chloride	100-44-7	2.06	OS	Eye, skin, and upper respiratory tract irritation	2A, probable carcinogen
Bromodichloromethane	75-27-4	70.0	RfD	Central nervous system disturbances, eye, skin, and respiratory tract irritation	Liver and kidney damage. 2B, possible carcinogen
Bromoethene (Vinyl Bromide)	593-60-2	3.00	RfC	Upper respiratory and eye irritation, listlessness, loss of reflexes, headache, and vertigo	Liver damage
Bromoform	75-25-2	90.9	IUR	Skin and upper respiratory tract irritation, fatigue, headache, dizziness, and vomiting, disturbances of hearing, vision, mental confusion, muscular weakness, collapse, hallucinations, tremors	Central nervous system depression, permanent brain injury, kidney damage
Bromomethane	74-83-9	5.00	RfC	Respiratory paralysis, cyanosis, peripheral vascular collapse, hypothermia	Peripheral nervous system impairment, central nervous system depression, coma
Butyl benzene	104-51-8			Affects endocrine system (glands and hormones)	Liver damage. 2B, possible carcinogen
Carbon disulfide	75-15-0	700	RfC	Headache, upper respiratory tract irritation and mucosa of the eyes	Liver damage
Carbon tetrachloride	56-23-5	100	RfC	Affects endocrine system (glands and hormones)	Liver damage. 2B, possible carcinogen
Chlorobenzene	108-90-7	70.0	RfD	Headache, upper respiratory tract irritation and mucosa of the eyes	Liver damage
Chloroethane	75-00-3	10,000	RfC	Rapid cooling and possible frostbite, stupor, eye irritation, stomach cramps, lack of coordination, dizziness	Liver and kidney damage
Chloroform	67-66-3	4.35	RfD	Fainting, vomiting, dizziness and salivation, increased intercranial pressure and nausea, fatigue and headache	Central nervous system impairment, liver damage, embryo / fetal damage, may result in death due to respiratory and cardiac arrhythmias and failure. 2B, possible carcinogen
Chloromethane	74-87-3	90.0	RfC	Tiredness, blurred vision, mental confusion, headache, changes in mood, incoordination, staggering gait, tremors, nausea, giddiness	Central nervous system impairment, liver, kidney, testicular damage, teratogenic effects, coma, death
cis-1,2-Dichloroethene	156-59-2	7.00	RfD	Eye irritation, dermatitis, nose, throat, lungs, and digestive system irritation	Drowsiness, confusion, slow heartbeat, heart damage, liver damage
cis-1,3-Dichloropropene	542-75-6	20.0	RfC	Eyes, upper respiratory, and skin irritation, depression of the central nervous system, headache, nausea, vomiting, dizziness	Central nervous system impairment, liver, kidneys and heart damage
Cyclohexane	110-82-7	6,000	RfC	Headache, dizziness, narcosis, dry throat, eye irritation	Central nervous system impairment, death
Dibromochloromethane	124-48-1	70.0	RfD	Skin, eye, mucous membrane and upper respiratory tract irritation, central nervous system functional	Liver and kidney damage

Table SM5: Reference values and their sources along with short-term and long-term health effects for specific VOCs measured by Summa canisters using the TO-15 method. CAS # is the unique chemical identification number assigned by the Chemical Abstracts Service. Each reference value (Ref. value) was obtained from the US Environmental Protection Agency's Integrated Risk Information System. The primary value was the Inhalation Reference Concentration (RfC). For chemicals that did not have an RfC value, the following were used in the order: Reference Dose (RfD), Cancer Inhalation Unit Risk (IUR), and Cancer Oral Slope (OS). To convert oral dose to inhalation dose, 70 kg bodyweight and 20 m^3/day respiratory rate were assumed. A risk of 10^{-4} was assumed in using the cancer slope factors.

**Chemical name**	**CAS #**	**Ref. value ($\mu g/m^3$)**	**Source of ref. value**	**Short-term health effects**	**Long-term health effects**
				disturbance, lung and cornea irritation	
Dichlorodifluoromethane(F-12)	75-71-8	700	RfD	Cardiac arrythmia, confusion, drowsiness, unconsciousness, frostbite, eye pain and redness	Cardiac sensitization
Dichlorotetrafluoroethane(F-114)	76-14-2			Cardiac arrythmia, confusion, drowsiness, unconsciousness, frostbite, eye pain and redness	Cardiac sensitization
Ethanol	64-17-5			Upper respiratory tract and eye irritation, liver damage, central nervous system depression, cancer	Liver damage, cancer
Ethyl Acetate	141-78-6	3,150	RfD	Eye and upper respiratory tract irritation, weakness, drowsiness, unconsciousness	Central nervous system depression, congestion of the liver and kidneys, and anemia with leukocytosis
Ethylbenzene	100-41-4	1,000	RfC	Upper respiratory tract irritation	Kidney damage (neuropathy), cochlear impairment. 2B, possible carcinogen
Heptane	142-82-5			Upper respiratory tract irritation, dullness, headache, dry skin, eye redness, abdominal cramps, burning sensation, nausea, vomiting	Central nervous system impairment, may have effects on the liver, and may cause chemical pneumonia
Hexachlorobutadiene	87-68-3	4.55	IUR	Development of hypotension, cardiac disease, chronic bronchitis, disturbance of nervous function	Kidney damage, cancer
Hexane	110-54-3	700	RfC	Eye irritation	Central nervous system impairment, peripheral neuropathy
Isopropylbenzene	98-82-8	400	RfC	Eyes, skin, and upper respiratory tract irritation	Central nervous system impairment. 2B, probable carcinogen
m&p-Xylene	179601-23-1	100	RfC	Unknown	Unknown
Methyl tert-butyl ether	1634-04-4	3,000	RfC	Upper respiratory tract irritation	Liver, nervous system, kidney damage, urinary system
Methylcyclohexane	108-87-2			Eye and skin irritant, lung and kidney damage, central nervous system impairment	Skin irritant
Methylene chloride	75-09-2	600	RfC	Carboxyhemoglobin, dizziness, nausea, tingling or numbness of fingers and toes, may cause problem with attention span and coordination, burning and redness of skin	Central nervous system impairment, liver cancer
Naphthalene	91-20-3	3.00	RfC	Upper respiratory tract irritation	Cataracts, hemolytic anemia. 2B, possible carcinogen
Nonane	111-84-2			Poor coordination, seizures, cough, passing out, dry skin, eye irritation, nausea, throwing up	Drowsiness, confusion, slow heartbeat
Octane	111-65-9			Nose, throat, and eye irritation, confusion, dizziness, drowsiness, headache, cough, labored breathing, nausea, passing out, and dry skin	Seizures
o-Xylene	95-47-6	100	RfC	Upper respiratory and eye irritation.	Central nervous system impairment
Propene (Propylene)	115-07-1			Asphyxia, upper respiratory tract irritation, skin and eye irritation, impaired judgement, disorientation, dizziness, decreased pain awareness, unconsciousness	Unknown
Propylbenzene	103-65-1			Irritation of the mucous membranes, eyes, nose, throat, and skin, depression of the central nervous system, headache, anorexia, muscular weakness, incoordination, nausea, vertigo, mental confusion and unconsciousness	Unknown

Table SM5: Reference values and their sources along with short-term and long-term health effects for specific VOCs measured by Summa canisters using the TO-15 method. CAS # is the unique chemical identification number assigned by the Chemical Abstracts Service. Each reference value (Ref. value) was obtained from the US Environmental Protection Agency's Integrated Risk Information System. The primary value was the Inhalation Reference Concentration (RfC). For chemicals that did not have an RfC value, the following were used in the order: Reference Dose (RfD), Cancer Inhalation Unit Risk (IUR), and Cancer Oral Slope (OS). To convert oral dose to inhalation dose, 70 kg bodyweight and 20 m^3/day respiratory rate were assumed. A risk of 10^{-4} was assumed in using the cancer slope factors.

**Chemical name**	**CAS #**	**Ref. value ($\mu g/m^3$)**	**Source of ref. value**	**Short-term health effects**	**Long-term health effects**
Sec-Butyl benzene	135-98-8			Headache, dizziness, weakness, and nausea, respiratory tract irritation	Potential eye damage
Styrene	100-42-5	1,000	RfC	Upper respiratory tract irritation	Central nervous system impairment, peripheral neuropathy
Tert-Butyl benzene	98-06-6			Headache, dizziness, weakness, nausea, eye, skin, and respiratory tract irritation	Acute lung injury
Tetrachloroethene	127-18-4	40.0	RfC	Eye, nose, and throat irritation, headache, drowsiness, and sleepiness	Central nervous system impairment. 2B, probable carcinogen
Tetrahydrofuran	109-99-9	2,000	RfC	Upper respiratory tract irritation	Central nervous system impairment, kidney damage. 2B, possible carcinogen
Toluene	108-88-3	5,000	RfC	Visual impairment	Female reproductive effects, loss of pregnancy
trans-1,2-Dichloroethene	156-60-5	70.0	RfD	Eye irritation, nausea, drowsiness, fatigue, vertigo	Central nervous system impairment
trans-1,3-Dichloropropene	542-75-6	20.0	RfC	Eyes, upper respiratory, and skin irritation, depression of the central nervous system, headache, nausea, vomiting, dizziness	Central nervous system impairment, liver, kidneys and heart damage
Trichloroethene	79-01-6	2.00	RfC	Dizziness, headache, sleepiness, nausea, confusion, blurred vision, weakness, altered motor skills	Central nervous system impairment, cognitive decrements, renal toxicity, cancer, and death
Trichlorofluoromethane(F-11)	75-69-4	1,050	RfD	Cardiac arrythmia, confusion, drowsiness, unconsciousness, frostbite, eye pain and redness	Cardiac sensitization
Trichlorotrifluoroethane(F-113)	76-13-1	105,000	RfD	Cardiac arrythmia, confusion, drowsiness, unconsciousness, frostbite, eye pain and redness	Cardiac sensitization
Vinyl acetate	108-05-4	200	RfC	Upper Respiratory tract, eye, and skin irritation	Central nervous system impairment. 2B, probable carcinogen
Vinyl chloride	75-01-4	100	RfC	Heart problems	Lung cancer, liver damage

Quality control data for specific VOCs

Overall quality control results were acceptable for duplicate and blank samples. For the 15 duplicate samples, the relative percent difference (RPD) of the hazard scores (HS_1 and HS_2) calculated as $RPD = |HS_1 - HS_2|/[(HS_1 + HS_2)/2] * 100\%$ had a mean of 23% and standard deviation of 32%. Supplemental Material Figure SM3 shows (A) the duplicate hazard scores themselves plotted against each other, and (B) a histogram of the RPD between hazard scores for duplicate samples. For the blank samples, hazard scores were calculated as a quality control check: hazard scores for 12/13 blank samples were missing, because 10/13 blank samples had no specific VOCs detected, and 2/13 blank samples had only trace amounts (7 ppb and 12 ppb) of ethanol (which has no reference value) detected. The remaining blank sample had a negligible hazard score (0.008) with trace amounts of acetone and ethanol detected (11 ppb each). Given that most (10/13) blank samples had no specific VOCs detected and the remaining 3 blank samples had only trace amounts of 1-2 specific VOCs detected, no blank corrections were necessary.

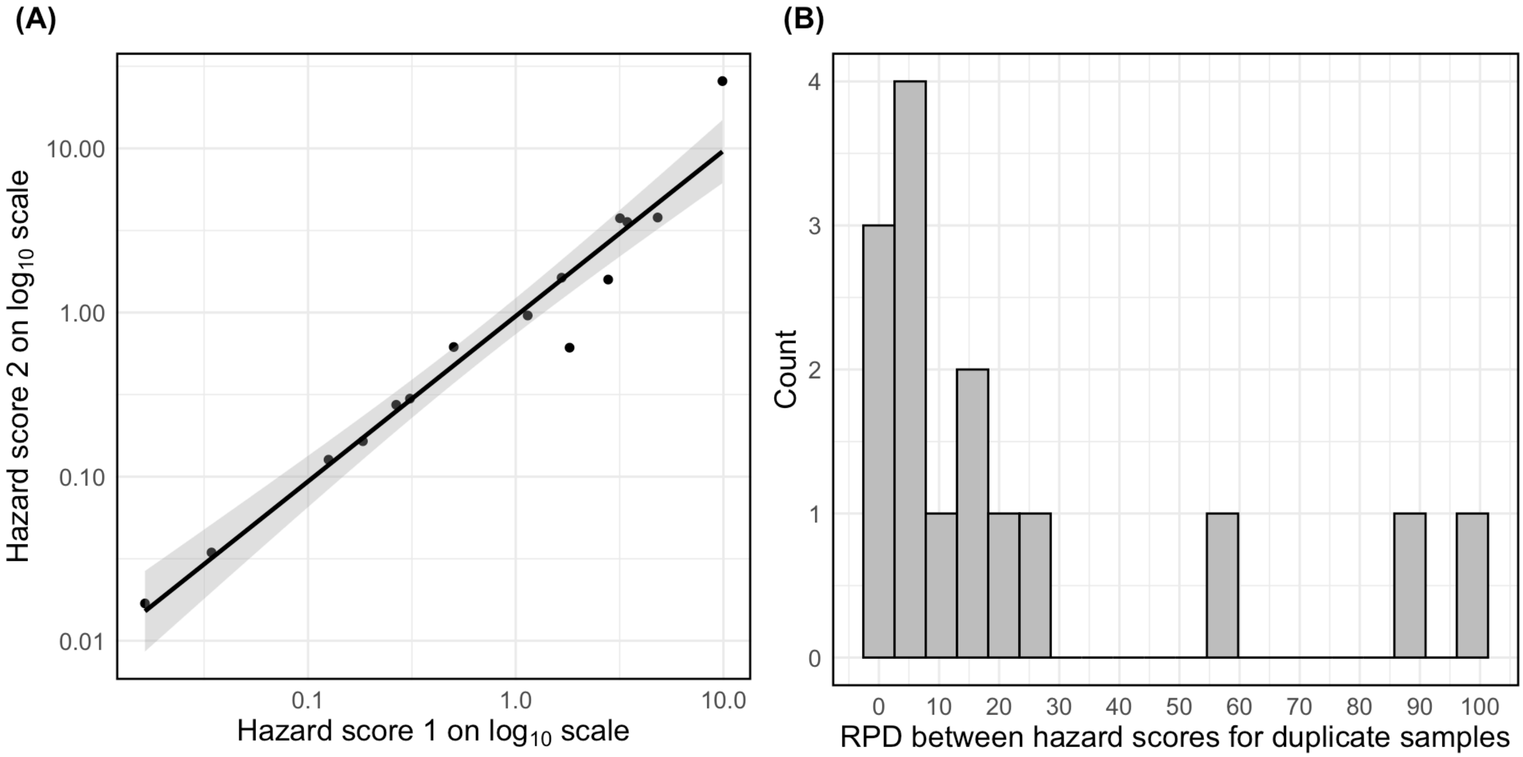

Figure SM3: Plots of quality control data for duplicate samples. (A) Comparison of hazard scores for duplicate samples on $\log_{10}$ scale. A regression line fit to the datapoints with 95% CI is shown, which aligns closely with the diagonal as expected. (B) Histogram of the relative percent difference (RPD) between hazard scores for duplicate samples.

Heatmaps of specific VOCs

Heatmap of specific VOCs measured with the TO-15 method in auto shops

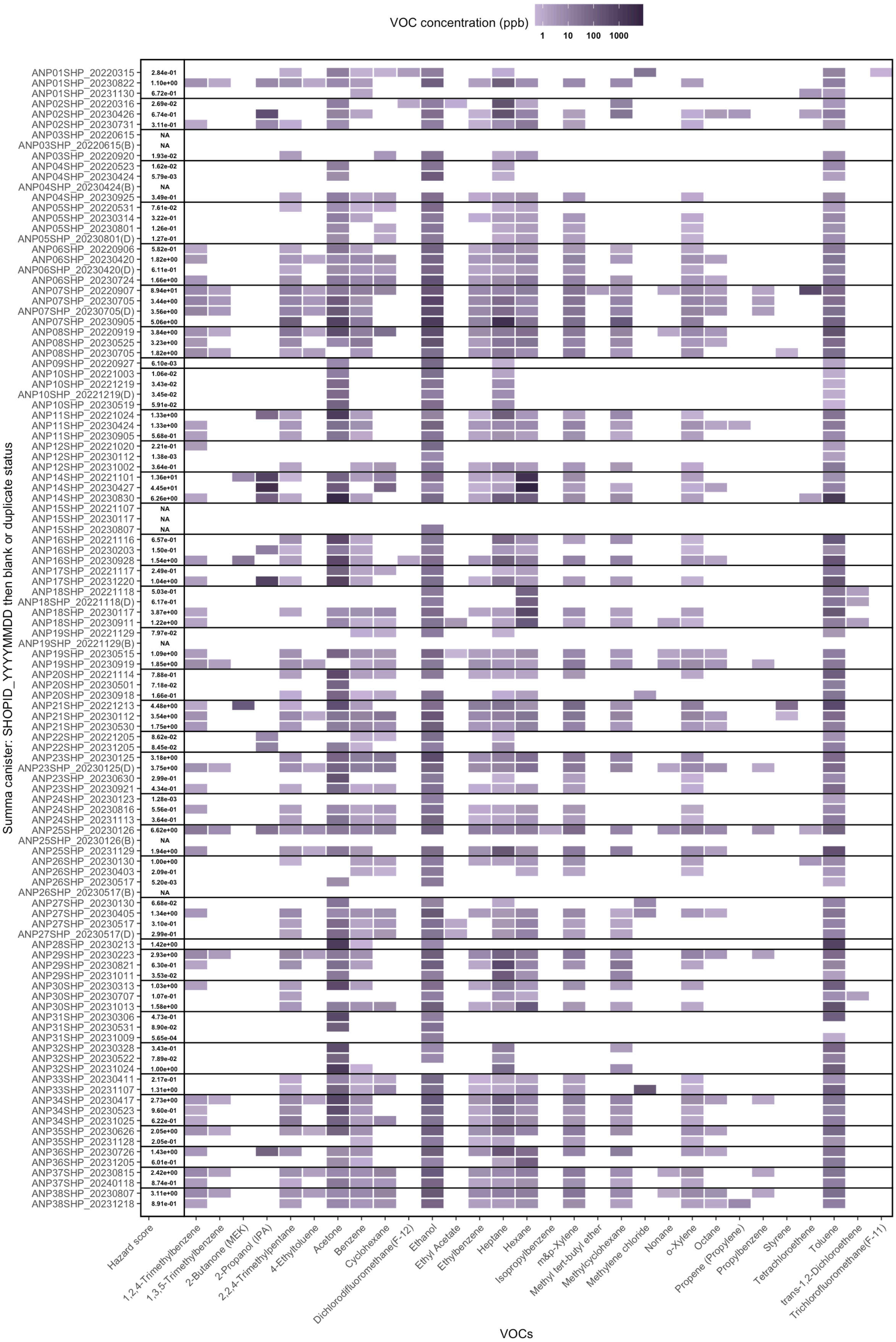

Figure SM4: Specific VOC concentrations (ppb) at each auto shop assessment, measured using Summa canisters and the US EPA TO-15 method, for VOCs that were detected in at least one auto shop. The hazard score, shown in the first column, is the sum of the ratios of each VOC's measured concentration to its reference value for each Summa canister. Each Summa canister is labeled by its shop ID, date in YYYYMMDD format, and blank (B) or duplicate (D) status.

Heatmap of specific VOCs measured with the TO-15 method in beauty shops

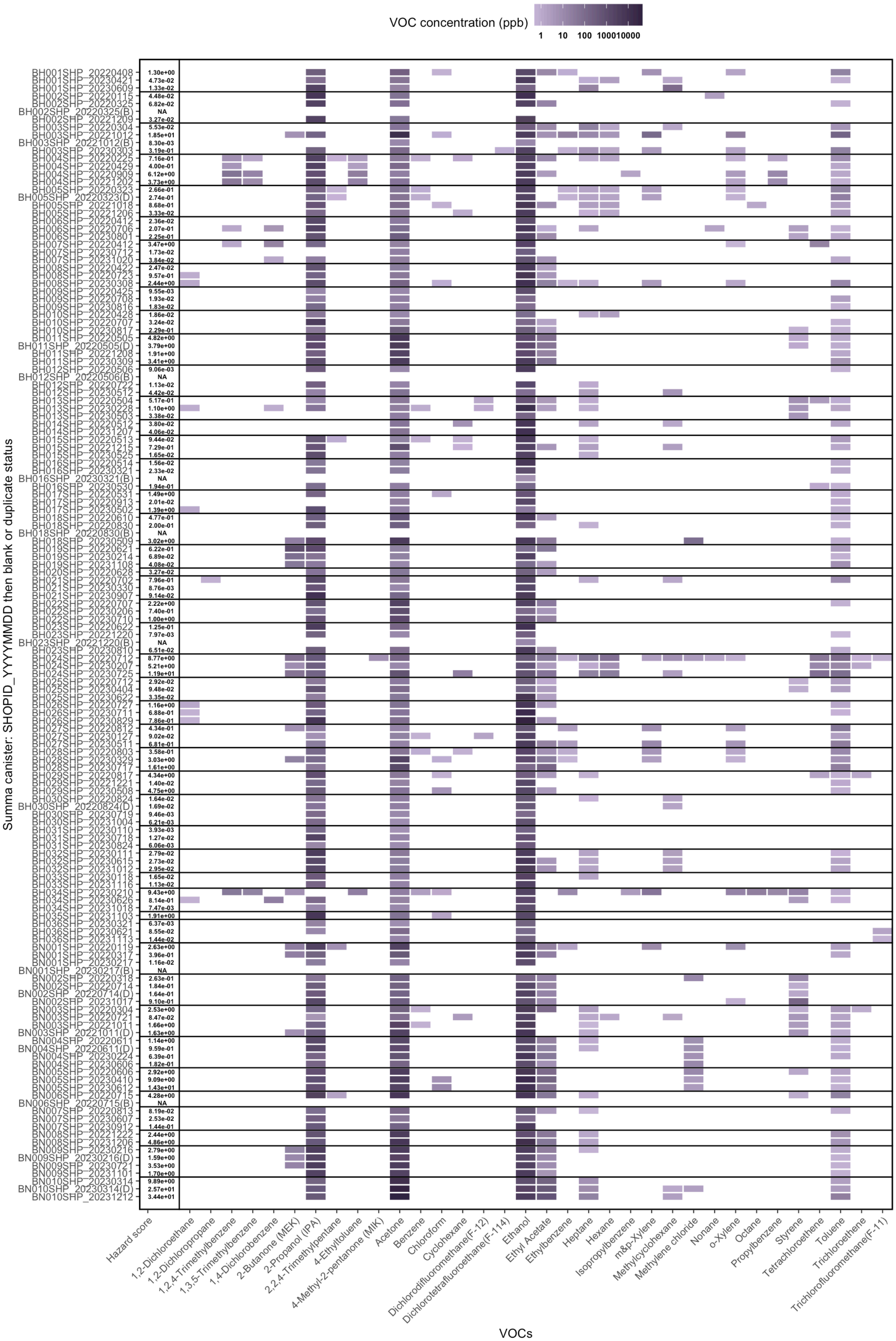

Figure SM5: Specific VOC concentrations (ppb) at each beauty shop assessment, measured using Summa canisters and the US EPA TO-15 method, for VOCs that were detected in at least one beauty shop. The hazard score, shown in the first column, is the sum of the ratios of each VOC's measured concentration to its reference value for each Summa canister. Each Summa canister is labeled by its shop ID, date in YYYYMMDD format, and blank (B) or duplicate (D) status.

Plots of data: hazard scores at each assessment

Figure SM6 shows violin plots of hazard score at each shop at each assessment for each intervention group. While this plot shows much variation at each assessment making it difficult to see a treatment effect, it also shows that many shop visits at both auto and beauty shops had air considered hazardous to human health, with hazard scores above 1.

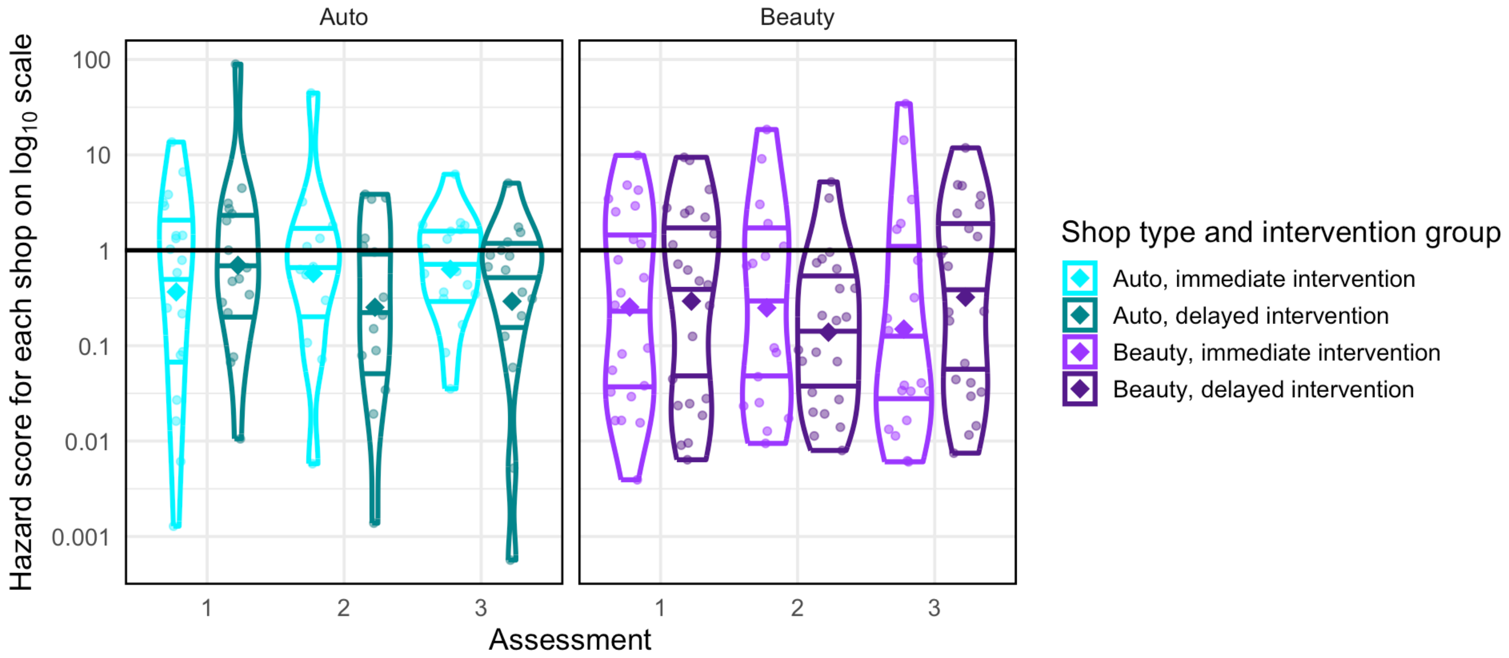

Figure SM6: Each datapoint in this plot represents the hazard score at one shop at each assessment (blank and duplicates have been removed). Horizontal lines in the violin plots represent the 25th percentile (Q1), 50th percentile (median), and 75th percentile (Q3). The diamond represents the geometric mean. The shape of the violin represents the relative frequency of data points at different values, based on a smoothed kernel density estimation, and the bounds correspond to the range over which the density is estimated (typically the range of the data). A black horizonal line at a hazard score of 1 indicates the threshold above which VOCs in the shop may be hazardous to human health.

Relationship between hazard scores and TVOCs and comparison to Ricklund et al. (2022)

To observe the relationship between our secondary and primary outcomes, we plotted hazard scores versus TVOCs (Figure SM7). As expected, shop visits with higher TVOCs tended to have higher hazard scores. The value of R^2 ranged from 0.22 to 0.41, indicating that only 22–41% of the variation in $\log_{10}$(hazard score) was explained by $\log_{10}$(TVOCs). Whereas beauty shops tended to have higher TVOCs than auto shops, they had a similar range of hazard scores (most shop assessments had hazard scores between 0.01 and 10).

Defining TVOCs as $TVOCs = \sum_{i=1}^{m} C_i$ where C_i is the concentration of the i-th chemical, and hazard score (HS) as $HS = \sum_{j=1}^{n<m} \frac{C_j}{ref_j}$ where ref_j is the reference concentration for the j-th chemical, and C_j is the concentration of the chemical for which a reference value exists, we might make the simple assumption that HS is proportional to TVOCs: $HS = \beta \cdot TVOCs$. Then $log_{10}(HS) = log_{10}(\beta) + log_{10}(TVOCs)$, and the slope on the $\log_{10}$–$\log_{10}$ plot would be 1. However, in reality the slope may differ from one because (1) not all chemicals in TVOCs were measured by the Summa canisters, and of those measured, not all had reference values (i.e., $n < m$), so they did not contribute to the HS, (2) chemicals that dominate a change in TVOCs may not necessarily dominate the HS (because they are not very toxic and have high reference values), and (3) some chemicals only contribute to the HS once their concentration has increased above the LOD. The slopes calculated for $log_{10}(HS)$ regressed on $log_{10}(TVOCs)$ were all consistent with (but could differ somewhat from) 1, with large 95% CIs (See Table SM6). For example, the estimated slope for beauty shops at assessment 1 was 1.15 with a 95% CI of (0.65, 1.65). Thus, the results from fitting our data were in line with our theoretical expectations.

Ricklund et al. (2022) measured TVOCs and hazard scores for three hairdressers in each of ten Swedish hair salons. To compare our TVOCs and hazard score results with theirs, we converted their TVOCs concentration from $\mu g/m^3$ *toluene equivalents* to *ppb isobutylene equivalents* as follows:

$$ppb_{isobutylene} = \frac{C_{toluene} \times 24.45}{MW_{toluene}} \times \frac{MW_{toluene}}{MW_{isobutylene}} = \frac{C_{toluene} \times 24.45}{MW_{isobutylene}}$$

where $ppb_{isobutylene}$ is the TVOC concentration in ppb (isobutylene equivalents), $C_{toluene}$ is the TVOC concentration in $\mu g/m^3$ (toluene equivalents), 24.45 is the molar volume of gas in L/mol at standard atmospheric temperature and pressure (25 °C and 1 atmosphere), and $MW_{isobutylene}$ is the molecular weight of isobutylene of 56.11 *g/mol*. Then, because Ricklund et al. (2022) had three correlated datapoints for each of their ten shops, we took the geometric mean TVOCs and the geometric mean hazard index for each shop. Next, we plotted their data on the $\log_{10}$–$\log_{10}$ scale (instead of on the original scale as in their paper – see last panel of Figure SM7).

The current study measured and defined TVOCs differently than Ricklund et al. (2022). Our TVOCs were defined as those with ionization energies that could be ionized by the 10.6 eV UV lamp in our PIDs. However, their TVOCs were defined as "the concentration corresponding to the summarized peak area of all compounds eluting between hexane and hexadecane in the gas chromatographic mass spectrums acquired from chemical analysis". The differences in detection methods and definitions likely contribute to why our beauty shop TVOCs were roughly an order of magnitude higher than theirs: our PID method detected a range of compounds with ionization energies less than or equal to 10.6 eV, potentially including a large concentration of light and highly reactive VOCs that Ricklund et al. (2022) might not capture as efficiently with gas chromatography mass spectrometry. These could include small alkenes or alcohols present in beauty shop environments. This comparison highlights the difficulty of comparing TVOCs between different studies: even though various studies measure TVOCs, the results are not directly comparable because the set of compounds each method detects is different.

The current study defined the hazard score (HS) similarly to how Ricklund et al. (2022) defined their hazard index (HI, which I will hereafter simply refer to as a hazard score): summing all the ratios formed by dividing the concentration of each chemical by its corresponding reference value, with a hazard score above 1 indicating air considered hazardous to human health. However, the current study and Ricklund et al. (2022) use different reference values and have different chemicals contributing to the final hazard score. The HS for beauty shops in our study ranged up to 34, whereas the HS in Ricklund et al. (2022) ranged up to 13 for individual workers (the max GM hazard score for a shop in their study was 7); however, most of our values of HS were in the range of the those from Ricklund et al. (2022).

Given the different methods of measuring TVOCs and different contributions to the hazard score, it is no surprise that our results do not exactly overlap with those from Ricklund et al. (2022). However, it is reassuring that even with the different methods, the slopes calculated for $\log_{10}$(hazard score) regressed on $\log_{10}$(TVOCs) from both our data and that of Ricklund et al. (2022) were all near 1, which aligns with our theoretical expectation that the HS is proportional to TVOCs (see Figure SM7 and Table SM6). Additionally, the value of R^2 for this regression from Ricklund et al. (2022) of 0.41 was close to the range of ours (0.22–0.40) for beauty shops as well.

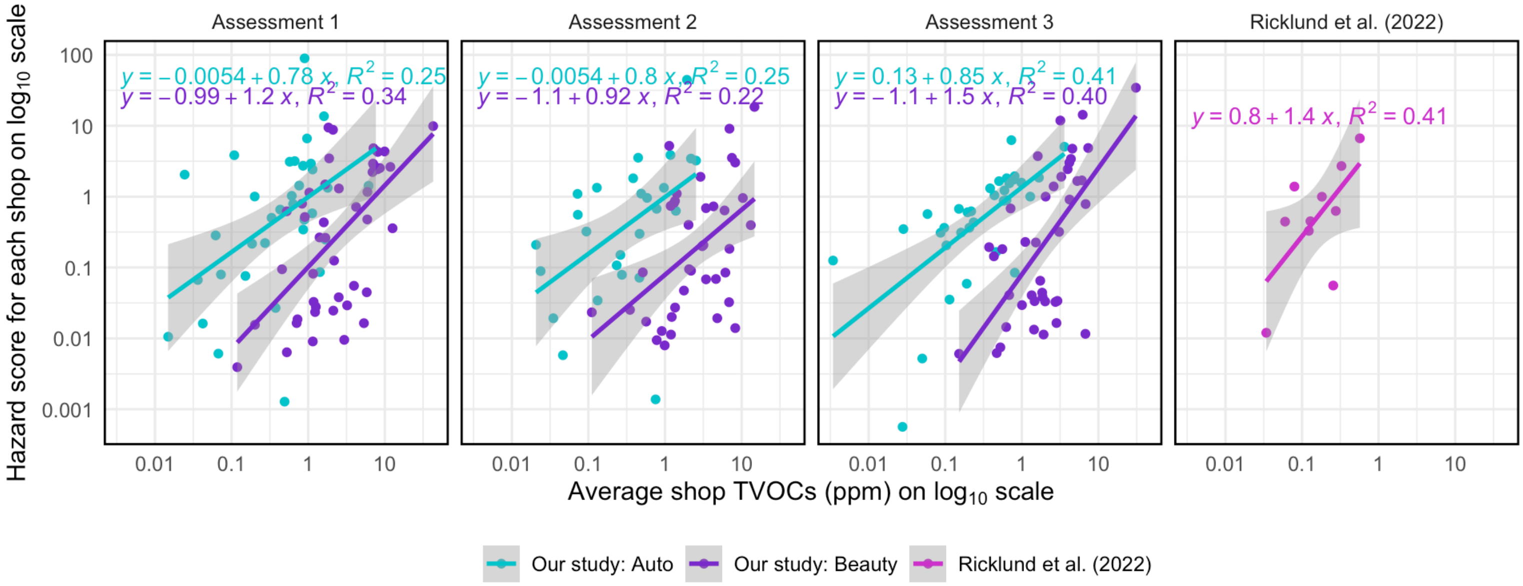

Figure SM7: Hazard scores versus average shop TVOC concentrations at each assessment. Average shop TVOC concentrations were calculated by taking the geometric mean of the TWA TVOCs for each shift of that shop for that assessment. Regression lines with 95% CIs are shown for each sector. The last panel shows the data from ten Swedish hair salons from Ricklund et al. (2022), as described above.

Table SM6: Summary of parameters for each model of $\log_{10}$(hazard score) on $\log_{10}$(TVOCs), by sector and assessment for our study, as well as for the data from ten Swedish hair salons from Ricklund et al. (2022).

Model	R^2	Slope (95% CI)
Our study: Auto, Assessment 1	0.25	0.78 (0.30, 1.26)
Our study: Auto, Assessment 2	0.25	0.80 (0.22, 1.38)
Our study: Auto, Assessment 3	0.41	0.85 (0.48, 1.23)
Our study: Beauty, Assessment 1	0.34	1.15 (0.65, 1.65)
Our study: Beauty, Assessment 2	0.22	0.92 (0.34, 1.50)
Our study: Beauty, Assessment 3	0.40	1.50 (0.90, 2.10)
Ricklund et al. (2022): Beauty	0.41	1.36 (0.02, 2.71)

Mixed models for hazard scores

Specifications for each model are given in Table SM7. The “Auto Adjusted” and “Beauty Adjusted” models are the main adjusted models discussed below. Because this data was so variable, many variables met the criteria for changing the treatment effect by 10% or more from either assessments 1-2 or 2-3: over 10 variables met this requirement for each sector (auto or beauty). However, often the results did not make sense (e.g., the inside air temperature at the beginning of the day would change the treatment effect by more than 10%, but its measurement at the end of the day would not). Given this, we just used the same set of covariates that we used for the primary outcome of TVOCs for the variant adjusted models.

Table SM8 presents estimated geometric means (GMs) of hazard scores and their 95% CIs along with estimated intervention effects for each model, and Figure SM8 plots the estimated GM hazard scores with their 95% CIs for each model. Table SM9 presents measures of the variation for each model.

Table SM7: Hazard score mixed model specifications for auto and beauty shops. Each model is of log-transformed hazard score on the fixed effects and random effects listed for that model. Values beyond baseline of possible post-treatment outcome variables (e.g., outside ventilation, air exchange rate) were not considered for inclusion in adjusted models.

Model	Reason	Fixed effects	Random effects
Auto Unadjusted		Intervention group<br>Assessment<br>Intervention group*Assessment	Shop
Auto Adjusted	Added all covariates that we expected might matter (same covariates as TVOCs adjusted model)	Intervention group<br>Assessment<br>Intervention group*Assessment<br>Shop-level baseline outside ventilation<br>Shift-level apparent temperature	Shop
Auto Adjusted (Variant)	Added same additional covariates used for adjusted variant model for TVOCs	Intervention group<br>Assessment<br>Intervention group*Assessment<br>Shop-level baseline outside ventilation<br>Shop-level self-report administration activity<br>Shift-level apparent temperature	Shop
Beauty Unadjusted		Intervention group<br>Assessment<br>Intervention group*Assessment	Shop
Beauty Adjusted	Added all covariates that we expected might matter (same covariates as TVOCs adjusted model)	Intervention group<br>Assessment<br>Intervention group*Assessment<br>Shop-level log-transformed baseline air exchange rate<br>Shop-level beauty shop type (hair and nails vs. hair only)	Shop
Beauty Adjusted (Variant)	Added same additional covariates used for adjusted variant model for TVOCs	Intervention group<br>Assessment<br>Intervention group*Assessment<br>Shop-level self-report administration activity<br>Shop-level self-report hair-processing activity	Shop

Table SM8: Estimated geometric means of hazard score and their 95% CIs for each combination of intervention group and assessment and for all intervention groups and all assessments, along with estimated intervention effects, from the mixed models for auto and beauty shops. Results have been back-transformed to the original scale from the log scale, which results in asymmetrical CIs and differences on the log scale have become ratios on the original scale. Three values of the estimated intervention effect are presented for each model. (1) The estimated intervention effect from assessments 1 & 2: (Immediate Time 2/Immediate Time 1) / (Delayed Time 2/Delayed Time 1). (2) The estimated intervention effect from assessments 2 & 3: (Delayed Time 3/Delayed Time 2) / (Immediate Time 3/Immediate Time 2). (3) The geometric mean of the prior two intervention effect estimates; this is the best estimate of the intervention effect for each model and what is presented in the main body of the paper. The estimated intervention effect should be evaluated by comparing to 1: a value less than 1 indicates that the intervention was effective in reducing the hazard score, a value greater than 1 indicates that the intervention may have increased the hazard score, and a 95% CI that includes 1 indicates that the effect of intervention was not statistically significant (so we cannot determine if it had a beneficial effect or not). Est. = Estimated; GM = geometric mean; CI: Confidence interval

Model	Intervention group	Assessment	Est. GM hazard score (95% CI)	Est. intervention effect from assessments 1 & 2 (95% CI)	Est. intervention effect from assessments 2 & 3 (95% CI)	Est. GM intervention effect from all assessments (95% CI)
Auto Unadjusted	Immediate	1	0.37 (0.14, 0.98)	3.45 (0.57, 20.72)	0.89 (0.15, 5.41)	1.75 (0.35, 8.69)
	Delayed	1	0.65 (0.22, 1.89)			
	Immediate	2	0.55 (0.17, 1.82)			
	Delayed	2	0.29 (0.09, 0.88)			
	Immediate	3	0.59 (0.21, 1.67)			
	Delayed	3	0.27 (0.09, 0.79)			
	All	All	0.43 (0.24, 0.77)			
Auto Adjusted	Immediate	1	0.20 (0.06, 0.71)	3.79 (0.57, 25.24)	0.89 (0.13, 6.09)	1.84 (0.34, 10.03)
	Delayed	1	0.53 (0.17, 1.59)			
	Immediate	2	0.35 (0.09, 1.39)			
	Delayed	2	0.24 (0.07, 0.75)			
	Immediate	3	0.38 (0.10, 1.41)			
	Delayed	3	0.23 (0.08, 0.69)			
	All	All	0.30 (0.14, 0.64)			
Auto Adjusted (Variant)	Immediate	1	0.20 (0.06, 0.68)	3.17 (0.46, 21.98)	0.70 (0.09, 5.10)	1.49 (0.26, 8.60)
	Delayed	1	0.51 (0.17, 1.54)			
	Immediate	2	0.29 (0.07, 1.21)			
	Delayed	2	0.23 (0.07, 0.75)			
	Immediate	3	0.35 (0.09, 1.32)			
	Delayed	3	0.20 (0.06, 0.62)			
	All	All	0.28 (0.13, 0.60)			
Beauty Unadjusted	Immediate	1	0.27 (0.10, 0.72)	2.08 (0.48, 9.09)	3.45 (0.76, 15.55)	2.68 (0.73, 9.90)
	Delayed	1	0.29 (0.11, 0.77)			
	Immediate	2	0.29 (0.09, 0.86)			
	Delayed	2	0.15 (0.06, 0.40)			
	Immediate	3	0.16 (0.06, 0.46)			
	Delayed	3	0.30 (0.11, 0.79)			
	All	All	0.23 (0.13, 0.42)			
Beauty Adjusted	Immediate	1	0.41 (0.16, 1.05)	1.95 (0.45, 8.44)	3.06 (0.68, 13.72)	2.44 (0.67, 8.97)
	Delayed	1	0.53 (0.20, 1.36)			
	Immediate	2	0.42 (0.14, 1.21)			
	Delayed	2	0.28 (0.10, 0.72)			
	Immediate	3	0.25 (0.09, 0.70)			
	Delayed	3	0.51 (0.20, 1.34)			
	All	All	0.38 (0.21, 0.70)			
Beauty Adjusted (Variant)	Immediate	1	0.18 (0.05, 0.62)	2.35 (0.46, 11.96)	4.27 (0.88, 20.80)	3.17 (0.77, 13.13)
	Delayed	1	0.20 (0.06, 0.67)			
	Immediate	2	0.23 (0.06, 0.83)			
	Delayed	2	0.11 (0.03, 0.34)			
	Immediate	3	0.12 (0.03, 0.43)			
	Delayed	3	0.25 (0.08, 0.73)			
	All	All	0.17 (0.08, 0.40)			

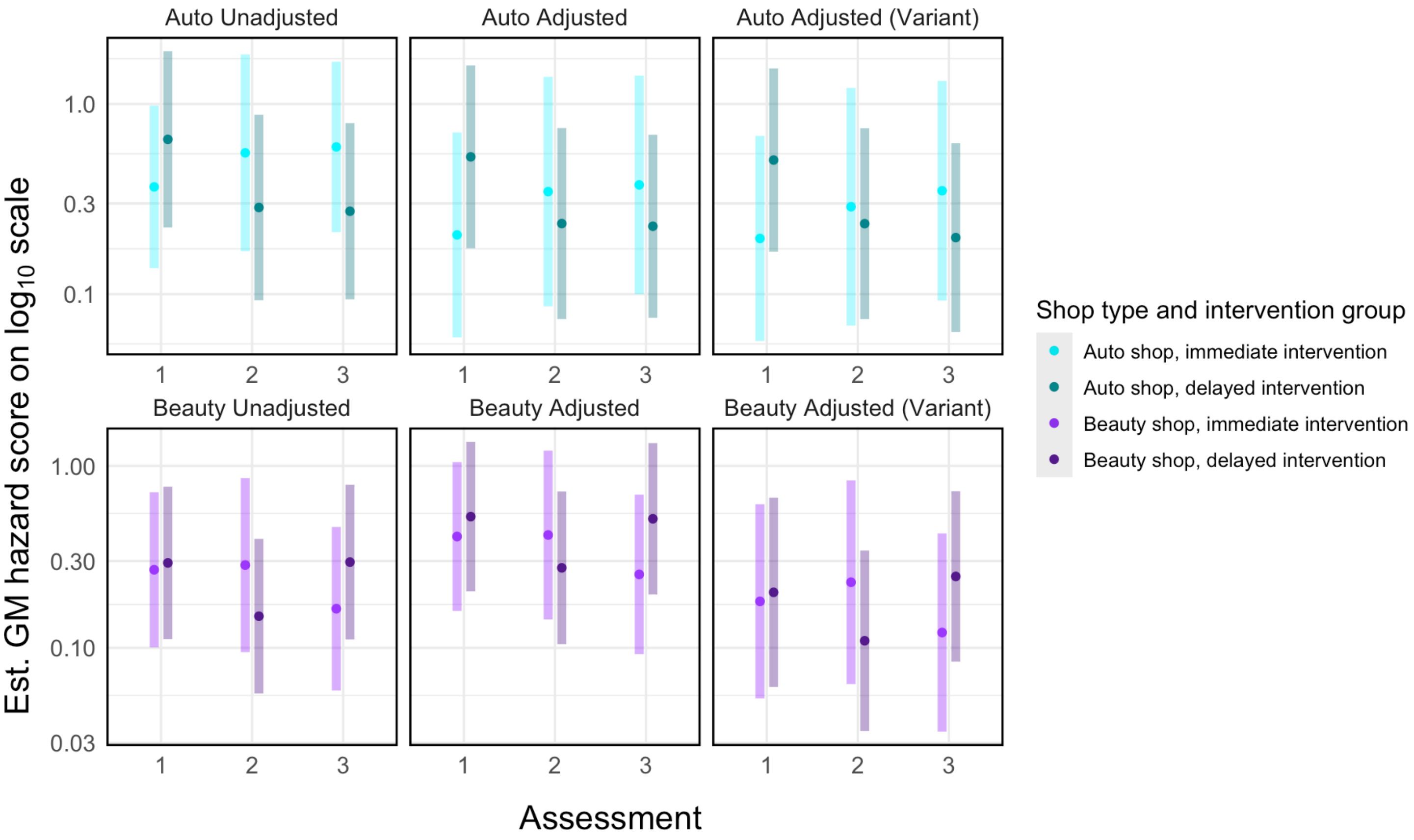

Figure SM8: Estimated geometric means of hazard score and their 95% CIs for each mixed model for auto and beauty shops. Note that these means have been plotted on the $\log_{10}$ scale.

Table SM9: Variance components for the hazard score mixed models for auto and beauty shops. The "Percent total variance" for the Shop row represents the shop-level ICC, which quantifies the proportion of total variance attributable to differences between shops.

Model	Groups	Variance	Standard deviation	Percent total variance
Auto Unadjusted	Shop	1.81	1.35	39
	Residual	2.80	1.67	61
Auto Adjusted	Shop	1.74	1.32	37
	Residual	2.91	1.71	63
Auto Adjusted (Variant)	Shop	1.71	1.31	37
	Residual	2.92	1.71	63
Beauty Unadjusted	Shop	2.74	1.66	51
	Residual	2.67	1.63	49
Beauty Adjusted	Shop	1.89	1.38	42
	Residual	2.65	1.63	58
Beauty Adjusted (Variant)	Shop	2.81	1.68	51
	Residual	2.67	1.63	49

The intervention did not statistically significantly reduce hazard scores for either auto or beauty shops (i.e., the 95% CI for the estimated intervention effect in Table SM8 included 1). Specifically, on average for the adjusted model for auto shops after intervention, there is a 84% non-statistically significant increase in hazard scores with 95% CI that goes from a 66% reduction to a 903% increase in hazard scores; on average for the adjusted model for beauty shops after intervention, there is a 144% non-statistically significant increase in hazard scores with 95% CI that goes from a 33% reduction to a 797% increase in hazard scores.

To compare hazard scores in different sectors, we used our adjusted models. Auto shops tended to have similar hazard scores to beauty shops: the estimated GM hazard score with 95% CI for all assessments in auto shops was 0.30 (0.14, 0.64); for beauty shops it was 0.38 (0.21, 0.70). Additionally, hair-and-nails beauty shops tended to have a factor of ~6 higher hazard scores than hair-only beauty shops: the estimated GM hazard score with 95% CI for all assessments in hair-and-nails shops was 0.97 (0.33, 2.80); for hair-only shops it was 0.15 (0.08, 0.27).

The large unexplained variability in the hazard score data made detecting a statistically significant intervention effect impossible. In the adjusted models, over half (63% for auto shops; 58% for beauty shops) of the variance was not accounted for by any term in the model, and the total variance in log(hazard score) for auto shops (4.6) was slightly higher than that for beauty shops (4.5; Table SM9).

Covariates

Outside TVOCs

The UA measurement team collected background measurements of the total volatile organic compounds (TVOCs) in air outside the shops. Generally, a background period was collected for each day during a shop assessment (so one shop assessment could have several background measurements), for 5-10 minutes prior to the start of workshifts at the shop. For each background period of outside TVOCs, the TWA outside TVOCs was calculated, just like the TWA of TVOCs was calculated for each workshift. Then because these data were log-normally distributed, we obtained the outside TVOCs for each shop assessment by taking the geometric average of the TWA TVOCs of all background periods within that shop assessment. Finally, because of their log-normal distribution, outside TVOCs were log-transformed for analysis.

Figure SM9 shows average TVOCs for each shop at each assessment versus the average outside TVOCs for each shop at each assessment (both on $\log_{10}$ scale). There is not much correlation between the two. Outside TVOCs are generally much lower than the TVOCs measured during the shop assessment, explaining why outside TVOCs do not appear to be correlated with the TVOCs during a shop assessment. Therefore, outside TVOCs were not included in the analyses.

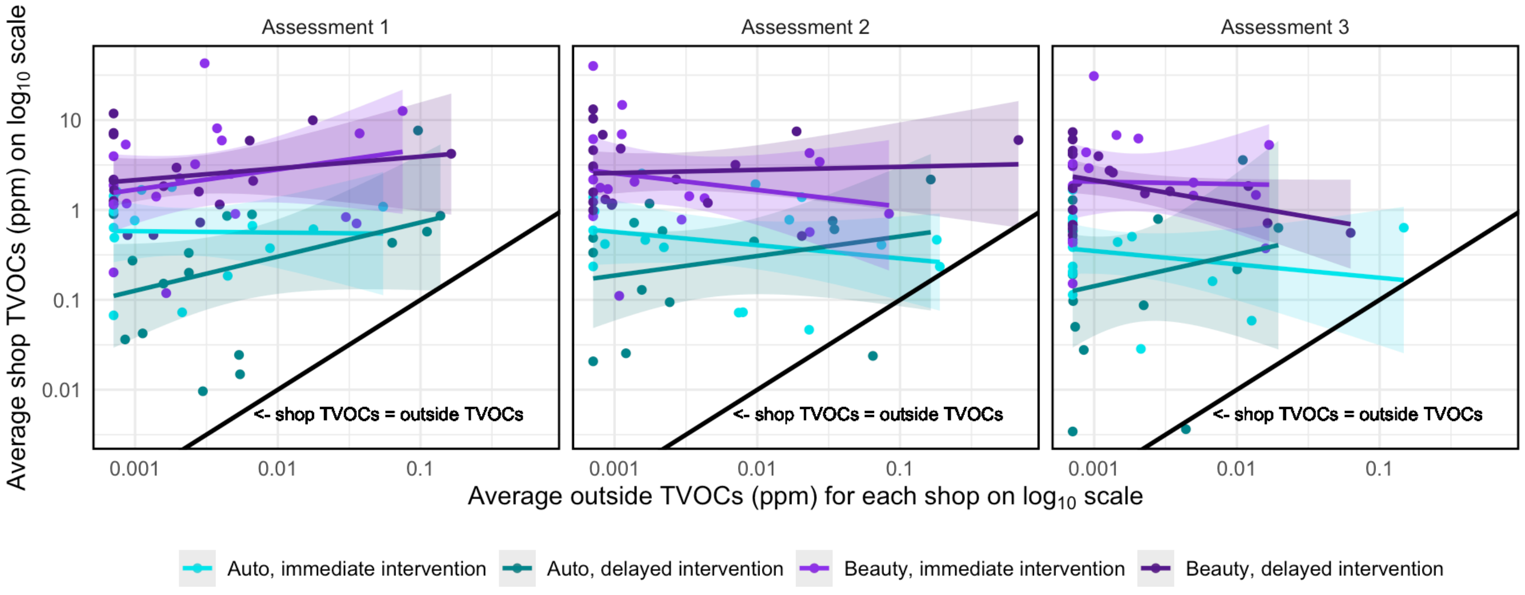

Figure SM9: Each datapoint in this plot represents average total volatile organic compounds (TVOCs) and average outside TVOCs at one shop at each assessment: for each shift, the TWA TVOCs was taken; then for each shop assessment the geometric average of the TWA TVOCs of all the shifts within that shop assessment was taken. Regression lines with 95% CIs are shown for each sector and intervention group. The black line in the lower right corner of the plot shows the line where shop TVOCs = outside TVOCs.

Air exchange rate

To account for differences in ventilation in beauty shops, air exchange rates were calculated from indoor CO_2 concentrations measured at each assessment using an Aranet4 HOME sensor (air exchange rate was missing for five shop assessments because of missing or obviously erroneous data). Air exchange rates at the beginning and end of day at each assessment were calculated as air changes per hour (ACH) from CO_2 data as follows:

$$ACH = \frac{6 \times 10^4 \;\; N\, G}{V\,\left(\bar{c}_{shop} - c_{outside}\right)}$$

where N is the number of people in the shop at the time of the measurement, G is the average CO_2 generation per person (assumed to be 0.3 L/min/person), V is the shop volume in m^3 (calculated from the measured shop dimensions), $\bar{c}_{shop}$ is the average CO_2 concentration in the shop in ppm (calculated from ten CO_2 measurements each a minute apart taken inside the shop near the center of the shop's main room five minutes after starting the CO_2 monitor), and $c_{outside}$ is the CO_2 concentration outside in ppm (taken from an outside measurement during the ten minutes before the sensor was taken indoors); see Batterman (2017). This calculation assumes steady state was reached and that the effect of anyone entering or leaving the room during the CO_2 measurements was minimal. Then the beginning and end of day values of ACH were averaged to get one air exchange rate for each shop at each assessment (if the beginning or end of day value was missing, it was assumed to be the non-missing value). Because of their skewed distribution, air exchange rates were log-transformed for analysis.

Apparent temperature

Figure SM10 shows average shop TVOCs versus the apparent temperature by sector and intervention group at each assessment. For auto shops, TVOCs tended to decrease as temperature increased except for in the delayed intervention group during assessment 2. For beauty shops, TVOCs and apparent temperature did not appear to be associated.

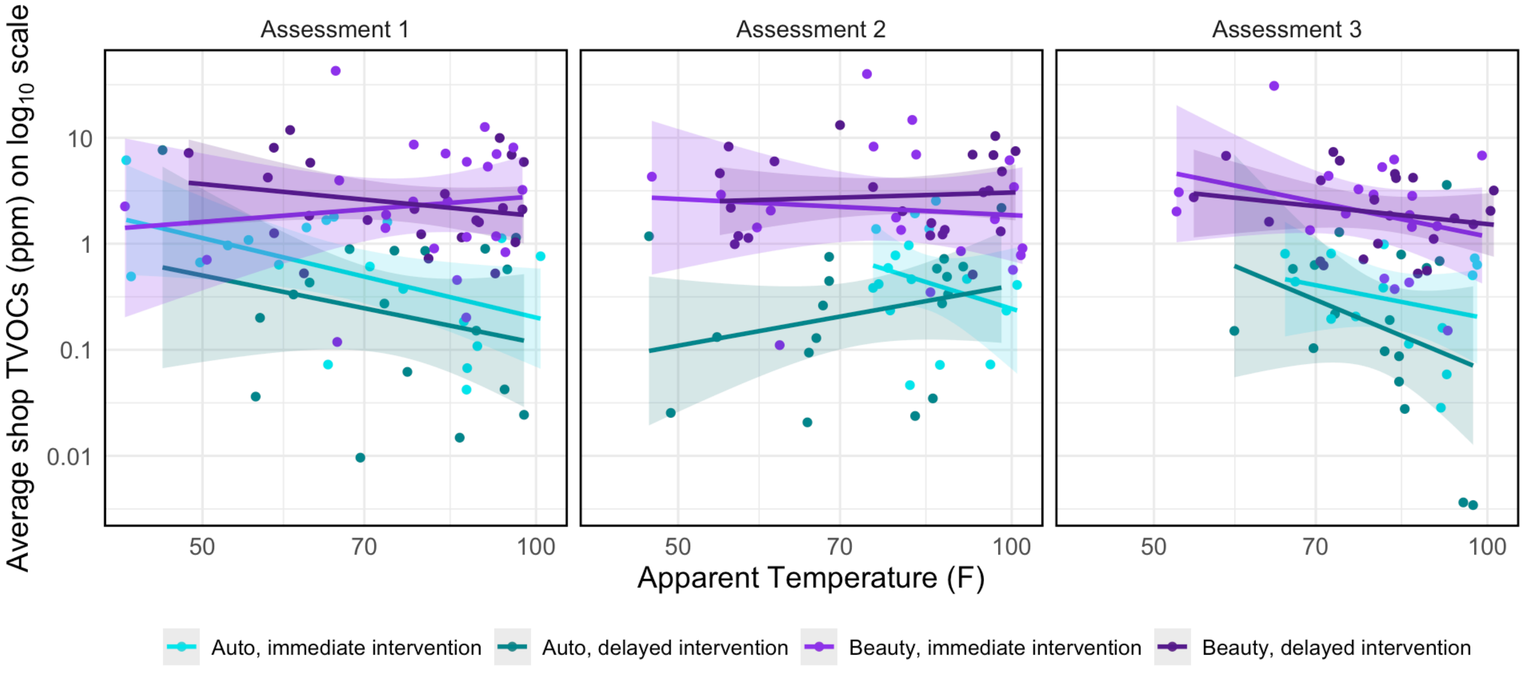

Figure SM10: Each datapoint in this plot represents TVOCs and apparent temperature data at one shop at each assessment: shop TVOCs are the geometric average of the TWA TVOCs for each shift of that shop for each assessment; apparent temperatures are the arithmetic average of the apparent temperatures for each shift of that shop for each assessment. Regression lines with 95% CIs are shown for each sector and intervention group.

Other data collected

Participants recorded activities and ventilation data on a written log (where they could write the time and check the categories of activities and ventilation conditions that occurred during their shift); however, accurate times were often missing, so UA staff entered data into REDCap for analysis as "Yes"/"No" for whether the activity or ventilation condition occurred during the shift. Auto activity categories were cleaning parts/tools, fluid services, mechanical/collision repair, painting, cleaning/disinfection, administrative, and other. Beauty activity categories were haircut/wash, hair styling, hair processing, nails, skin care, cleaning/disinfecting, administrative, and other. For both sectors, ventilation categories were central AC, swamp cooler, minisplit/wall AC unit, desk/floor fan, ceiling fan, open door/window, and local exhaust; additionally, auto shops included the category of outside ventilation. Self-report activity and ventilation data were considered as covariates at both the shift and shop levels; to aggregate this data to the shop level, the answer was "Yes" for a category if any participant at that shop assessment answered "Yes" for that category.

Shop-level outside ventilation (auto only)

Figure SM11 shows average shop TVOCs versus whether an auto shop was using outside ventilation (i.e., workers performing duties outdoors) at each assessment for each intervention group. As expected, shops that used outside ventilation during an assessment tended to have lower TVOCs; however, there is a lot of variation. This plot also shows the imbalance in outside ventilation at baseline (Assessment 1), with only one shop in the immediate intervention group using outside ventilation then, but seven shops in the delayed intervention group using outside ventilation then.

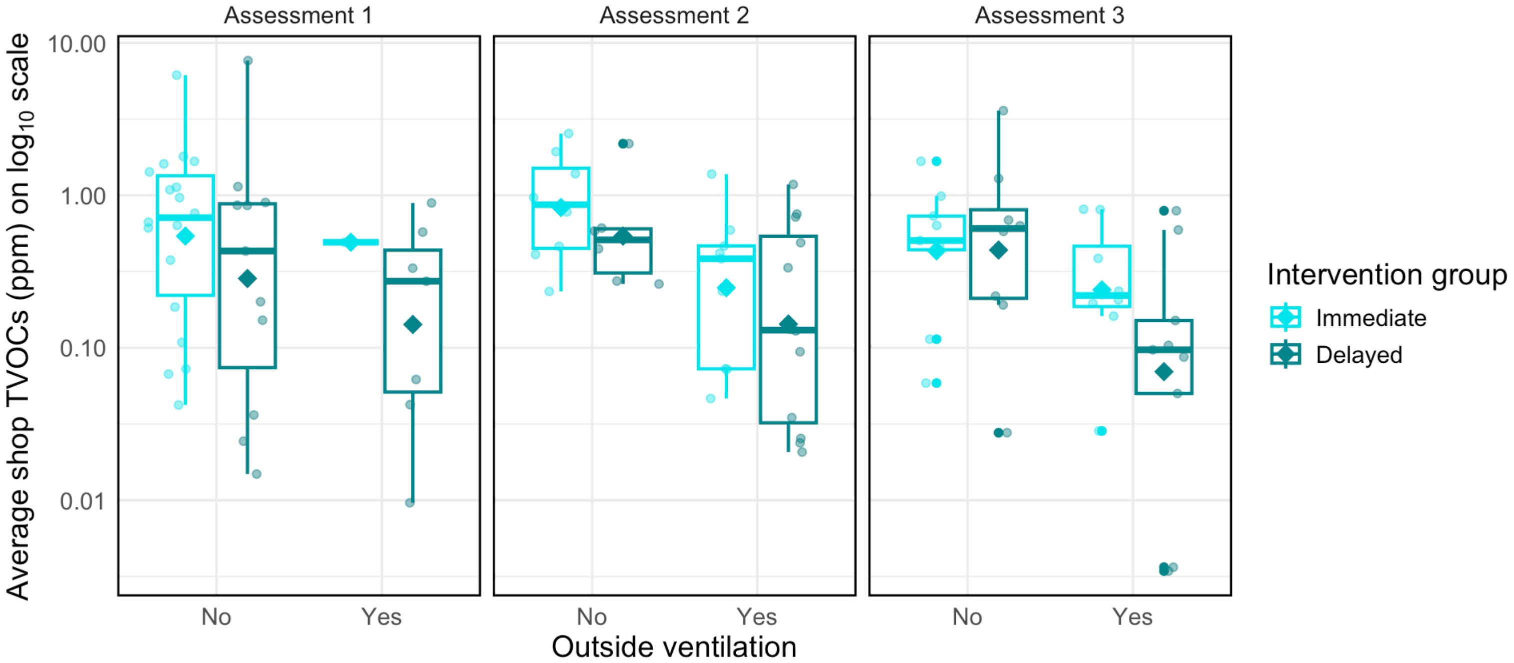

Figure SM11: Each datapoint in this plot represents TVOCs data at one shop at each assessment: it is the geometric mean of the TWA TVOCs for each shift of that shop an assessment. The boxplots represent the 25th percentile (Q1), 50th percentile (median), and 75th percentile (Q3); the diamond represents the geometric mean.

Beauty shop type (hair and nails vs. hair only)

Figure SM12 shows average shop TVOCs versus beauty shop type for each intervention group at each assessment. Hair and nails shops tend to have higher TVOCs than hair only shops, but there is a lot of variation.

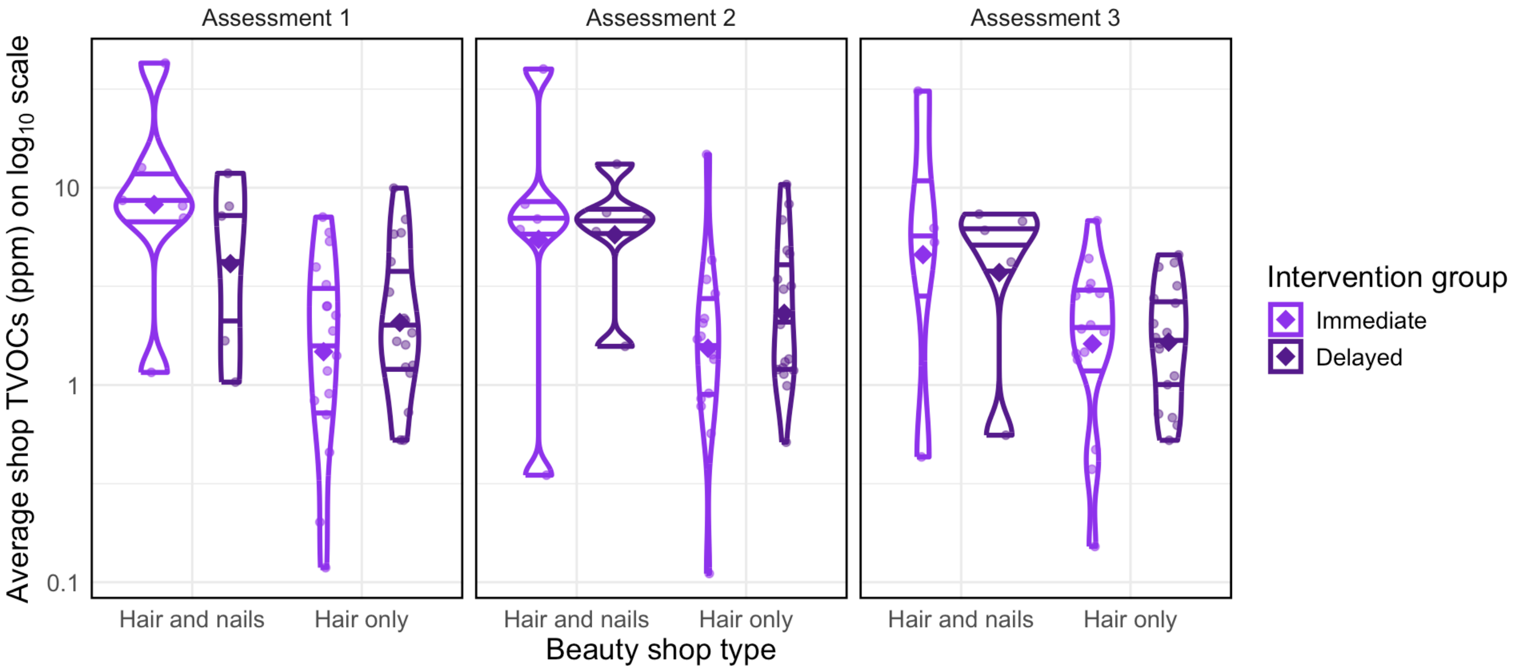

Figure SM12: Each datapoint in this plot represents TVOCs data at one shop at each assessment: it is the geometric mean of the TWA TVOCs for each shift of that shop an assessment. Horizontal lines in the violin plots represent the 25th percentile (Q1), 50th percentile (median), and 75th percentile (Q3). The diamond represents the geometric mean. The shape of the violin represents the relative frequency of data points at different values, based on a smoothed kernel density estimation, and the bounds correspond to the range over which the density is estimated (typically the range of the data).

Shop-level self-report administration activity

Figure SM13 shows average shop TVOCs versus shop-level self-report administration activity for each sector and intervention group, for each assessment. Shop-level self-report administration activity at a shop assessment was “Yes” if any worker at any shift at that shop during that assessment reported performing an administration activity during their shift.

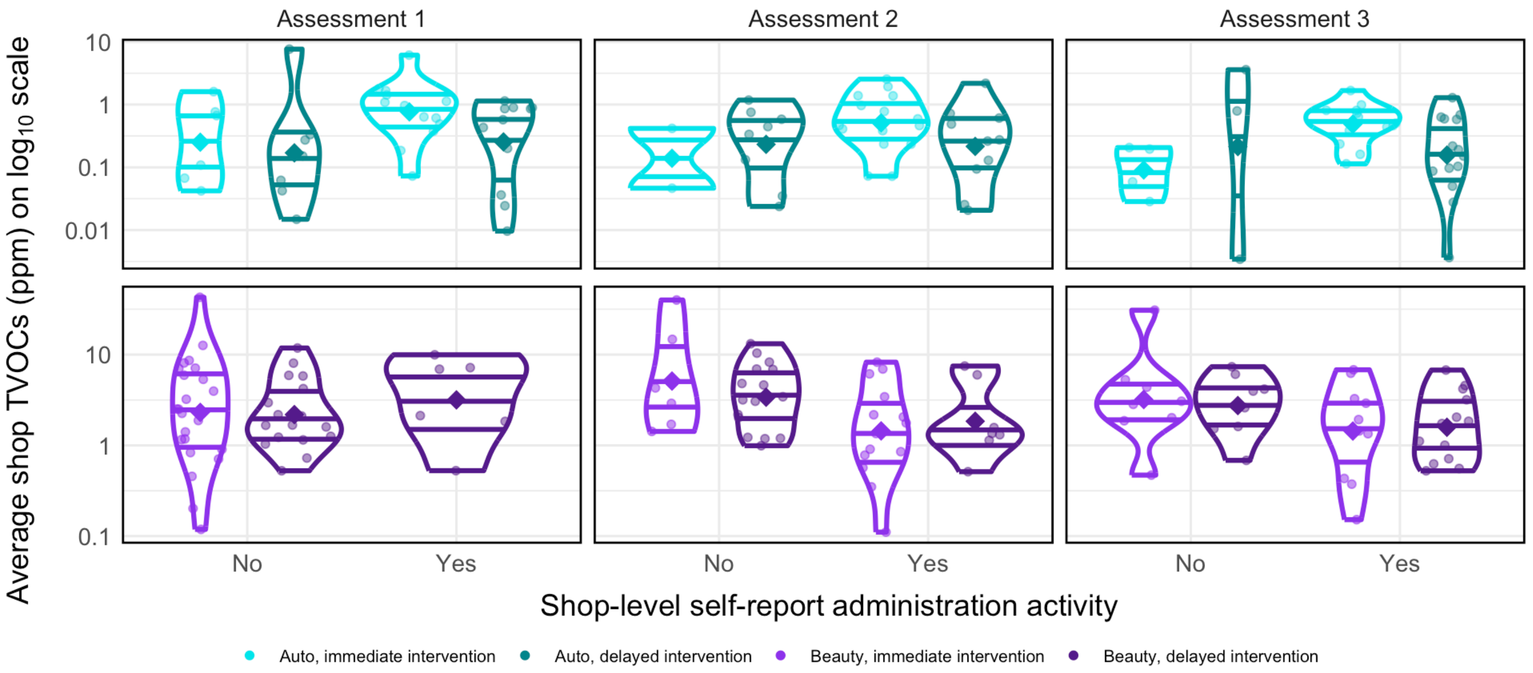

Figure SM13: Each datapoint in this plot represents TVOCs data at one shop at each assessment: it is the geometric mean of the TWA TVOCs for each shift of that shop an assessment. Horizontal lines in the violin plots represent the 25th percentile (Q1), 50th percentile (median), and 75th percentile (Q3). The diamond represents the geometric mean. The shape of the violin represents the relative frequency of data points at different values, based on a smoothed kernel density estimation, and the bounds correspond to the range over which the density is estimated (typically the range of the data).

Shop-level self-report hair-processing activity (beauty only)

Figure SM14 shows average shop TVOCs versus shop-level self-report hair-processing activity for each beauty shop intervention group, for each assessment. Shop-level self-report hair-processing activity at a shop assessment was “Yes” if any worker at any shift at that shop during that assessment reported performing a hair-processing activity during their shift.

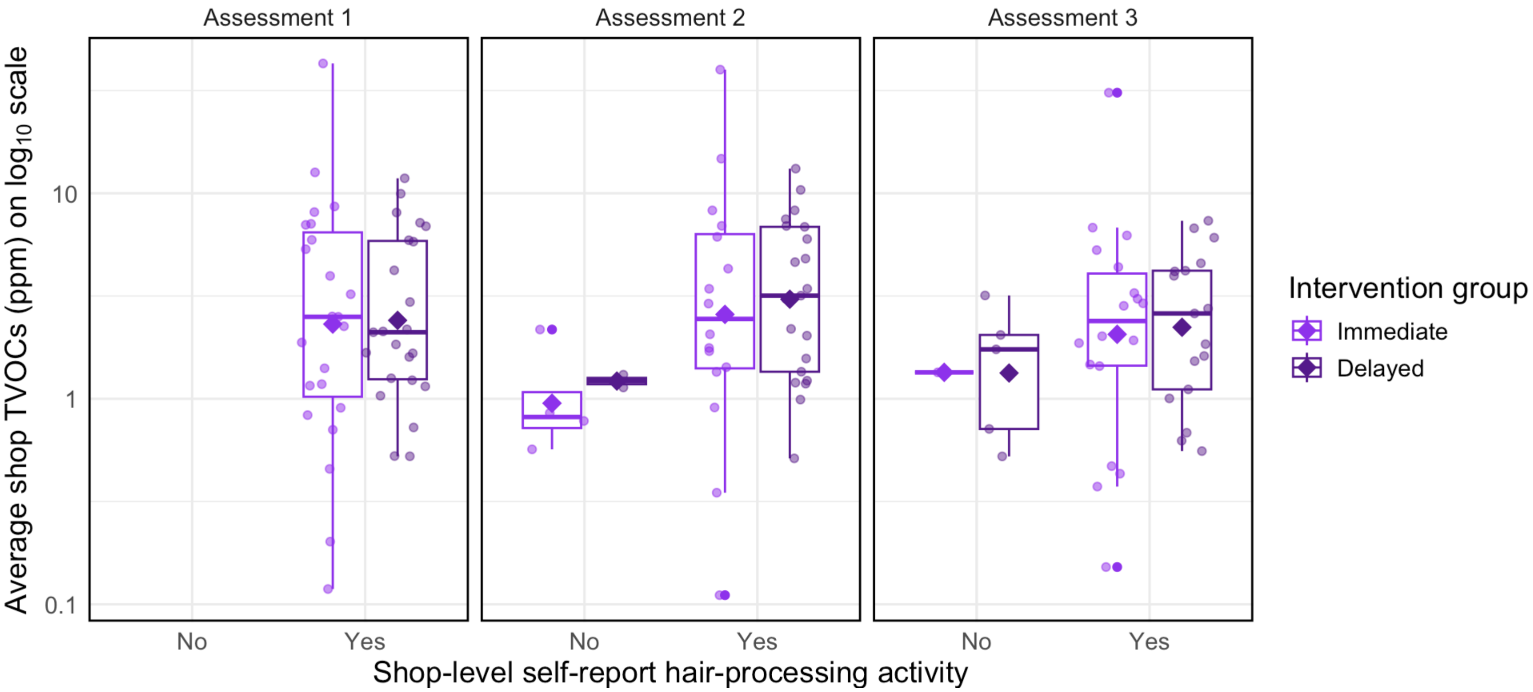

Figure SM14: Each datapoint in this plot represents TVOCs data at one shop at each assessment: it is the geometric mean of the TWA TVOCs for each shift of that shop an assessment. Boxplots represent the 25th percentile (Q1), 50th percentile (median), and 75th percentile (Q3); the diamond represents the geometric mean.

Versions and references for R and packages

This section is included because knowing the versions of R and packages is important for reproducible research, and to give credit to the developers of the packages.

Versions of R and packages used in this report from the sessionInfo() command:

```
## R version 4.4.1 (2024-06-14)
## Platform: aarch64-apple-darwin20
## Running under: macOS Ventura 13.6.7
##
## Matrix products: default
## BLAS:   /Library/Frameworks/R.framework/Versions/4.4-arm64/Resources/lib/libRblas.0.dylib
## LAPACK: /Library/Frameworks/R.framework/Versions/4.4-arm64/Resources/lib/libRlapack.dylib;  LAPACK version 3.12.0
##
## locale:
## [1] en_US.UTF-8/en_US.UTF-8/en_US.UTF-8/C/en_US.UTF-8/en_US.UTF-8
##
## time zone: America/Phoenix
## tzcode source: internal
##
## attached base packages:
## [1] stats     graphics  grDevices utils     datasets  methods   base
##
## other attached packages:
##  [1] forcats_1.0.0      stringr_1.5.1      dplyr_1.1.4       purrr_1.0.2
```

```
##  [5] readr_2.1.5        tidyr_1.3.1        tibble_3.2.1       tidyverse_2.0.0
##  [9] simplecolors_0.1.2 patchwork_1.3.0    cowplot_1.1.3      ggforce_0.4.2
## [13] htmltools_0.5.8.1  glue_1.7.0         rsvg_2.6.0         DiagrammeRsvg_0.1
## [17] DiagrammeR_1.0.11  Hmisc_5.1-3        emmeans_1.10.4     lmerTest_3.1-3
## [21] lme4_1.1-35.5      Matrix_1.7-0       scales_1.3.0       lubridate_1.9.3
## [25] naniar_1.1.0       arsenal_3.6.3      gt_0.11.0          gtsummary_2.0.2
## [29] kableExtra_1.4.0   knitr_1.48         ggpmisc_0.6.0      ggpp_0.5.8-1
## [33] ggplot2_3.5.1
##
## loaded via a namespace (and not attached):
##  [1] RColorBrewer_1.1-3 rstudioapi_0.16.0  jsonlite_1.8.8
##  [4] magrittr_2.0.3     TH.data_1.1-2      estimability_1.5.1
##  [7] farver_2.1.2       cardx_0.2.1        nloptr_2.1.1
## [10] rmarkdown_2.28     ragg_1.3.2         vctrs_0.6.5
## [13] minqa_1.2.8        base64enc_0.1-3    polynom_1.4-1
## [16] curl_5.2.2         broom_1.0.6        Formula_1.2-5
## [19] sass_0.4.9         bslib_0.8.0        htmlwidgets_1.6.4
## [22] pbkrtest_0.5.3     sandwich_3.1-1     zoo_1.8-12
## [25] cachem_1.1.0       commonmark_1.9.1   lifecycle_1.0.4
## [28] pkgconfig_2.0.3    R6_2.5.1           fastmap_1.2.0
## [31] digest_0.6.37      numDeriv_2016.8-1.1 colorspace_2.1-1
## [34] confintr_1.0.2     textshaping_0.4.0  labeling_0.4.3
## [37] fansi_1.0.6        timechange_0.3.0   mgcv_1.9-1
## [40] polyclip_1.10-7    compiler_4.4.1     bit64_4.0.5
## [43] withr_3.0.1        htmlTable_2.4.3    backports_1.5.0
## [46] highr_0.11         MASS_7.3-61        quantreg_5.98
## [49] tools_4.4.1        foreign_0.8-87     visdat_0.6.0
## [52] nnet_7.3-19        nlme_3.1-166       grid_4.4.1
## [55] checkmate_2.3.2    cluster_2.1.6      generics_0.1.3
## [58] gtable_0.3.5       tzdb_0.4.0         data.table_1.16.0
## [61] hms_1.1.3          xml2_1.3.6         utf8_1.2.4
## [64] pillar_1.9.0       markdown_1.13      vroom_1.6.5
## [67] splines_4.4.1      tweenr_2.0.3       lattice_0.22-6
## [70] survival_3.7-0     bit_4.0.5          SparseM_1.84-2
## [73] tidyselect_1.2.1   gridExtra_2.3      V8_5.0.0
## [76] bookdown_0.40      svglite_2.1.3      xfun_0.47
## [79] visNetwork_2.1.2   stringi_1.8.4      yaml_2.3.10
## [82] boot_1.3-31        evaluate_0.24.0    codetools_0.2-20
## [85] cli_3.6.3          rpart_4.1.23       systemfonts_1.1.0
## [88] munsell_0.5.1      jquerylib_0.1.4    Rcpp_1.0.13
## [91] parallel_4.4.1     MatrixModels_0.5-3 viridisLite_0.4.2
## [94] mvtnorm_1.3-1      crayon_1.5.3       rlang_1.1.4
## [97] multcomp_1.4-26    cards_0.2.2
```

References for R and packages used in this report from the report::cite_packages() command:

- Aphalo P (2024). *ggpmisc: Miscellaneous Extensions to 'ggplot2'*. R package version 0.6.0, https://CRAN.R-project.org/package=ggpmisc.
- Aphalo P (2024). *ggpp: Grammar Extensions to 'ggplot2'*. R package version 0.5.8-1, https://CRAN.R-project.org/package=ggpp.
- Bates D, Mächler M, Bolker B, Walker S (2015). "Fitting Linear Mixed-Effects Models Using lme4." *Journal of Statistical Software*, *67*(1), 1-48. doi:10.18637/jss.v067.i01 https://doi.org/10.18637/jss.v067.i01.

- Bates D, Maechler M, Jagan M (2024). *Matrix: Sparse and Dense Matrix Classes and Methods*. R package version 1.7-0, https://CRAN.R-project.org/package=Matrix.
- Cheng J, Sievert C, Schloerke B, Chang W, Xie Y, Allen J (2024). *htmltools: Tools for HTML*. R package version 0.5.8.1, https://CRAN.R-project.org/package=htmltools.
- Grolemund G, Wickham H (2011). "Dates and Times Made Easy with lubridate." *Journal of Statistical Software*, *40*(3), 1-25. https://www.jstatsoft.org/v40/i03/.
- Harrell Jr F (2024). *Hmisc: Harrell Miscellaneous*. R package version 5.1-3, https://CRAN.R-project.org/package=Hmisc.
- Heinzen E, Sinnwell J, Atkinson E, Gunderson T, Dougherty G (2021). *arsenal: An Arsenal of 'R' Functions for Large-Scale Statistical Summaries*. R package version 3.6.3, https://CRAN.R-project.org/package=arsenal.
- Hester J, Bryan J (2024). *glue: Interpreted String Literals*. R package version 1.7.0, https://CRAN.R-project.org/package=glue.
- Iannone R (2016). *DiagrammeRsvg: Export DiagrammeR Graphviz Graphs as SVG*. R package version 0.1, https://CRAN.R-project.org/package=DiagrammeRsvg.
- Iannone R, Cheng J, Schloerke B, Hughes E, Lauer A, Seo J, Brevoort K, Roy O (2024). *gt: Easily Create Presentation-Ready Display Tables*. R package version 0.11.0, https://CRAN.R-project.org/package=gt.
- Iannone R, Roy O (2024). *DiagrammeR: Graph/Network Visualization*. R package version 1.0.11, https://CRAN.R-project.org/package=DiagrammeR.
- Kuznetsova A, Brockhoff PB, Christensen RHB (2017). "lmerTest Package: Tests in Linear Mixed Effects Models." *Journal of Statistical Software*, *82*(13), 1-26. doi:10.18637/jss.v082.i13 https://doi.org/10.18637/jss.v082.i13.
- Lenth R (2024). *emmeans: Estimated Marginal Means, aka Least-Squares Means*. R package version 1.10.4, https://CRAN.R-project.org/package=emmeans.
- Müller K, Wickham H (2023). *tibble: Simple Data Frames*. R package version 3.2.1, https://CRAN.R-project.org/package=tibble.
- Ooms J (2023). *rsvg: Render SVG Images into PDF, PNG, (Encapsulated) PostScript, or Bitmap Arrays*. R package version 2.6.0, https://CRAN.R-project.org/package=rsvg.
- Pedersen T (2024). *ggforce: Accelerating 'ggplot2'*. R package version 0.4.2, https://CRAN.R-project.org/package=ggforce.
- Pedersen T (2024). *patchwork: The Composer of Plots*. R package version 1.3.0, https://CRAN.R-project.org/package=patchwork.
- R Core Team (2024). *R: A Language and Environment for Statistical Computing*. R Foundation for Statistical Computing, Vienna, Austria. https://www.R-project.org/.
- Riley J (2023). *simplecolors: Access Color Names Using a Standardized Nomenclature*. R package version 0.1.2, https://CRAN.R-project.org/package=simplecolors.
- Sjoberg D, Whiting K, Curry M, Lavery J, Larmarange J (2021). "Reproducible Summary Tables with the gtsummary Package." *The R Journal*, *13*, 570-580. doi:10.32614/RJ-2021-053 https://doi.org/10.32614/RJ-2021-053, https://doi.org/10.32614/RJ-2021-053.
- Tierney N, Cook D (2023). "Expanding Tidy Data Principles to Facilitate Missing Data Exploration, Visualization and Assessment of Imputations." *Journal of Statistical Software*, *105*(7), 1-31. doi:10.18637/jss.v105.i07 https://doi.org/10.18637/jss.v105.i07.
- Wickham H (2016). *ggplot2: Elegant Graphics for Data Analysis*. Springer-Verlag New York. ISBN 978-3-319-24277-4, https://ggplot2.tidyverse.org.
- Wickham H (2023). *forcats: Tools for Working with Categorical Variables (Factors)*. R package version 1.0.0, https://CRAN.R-project.org/package=forcats.
- Wickham H (2023). *stringr: Simple, Consistent Wrappers for Common String Operations*. R package version 1.5.1, https://CRAN.R-project.org/package=stringr.
- Wickham H, Averick M, Bryan J, Chang W, McGowan LD, François R, Grolemund G, Hayes A, Henry L, Hester J, Kuhn M, Pedersen TL, Miller E, Bache SM, Müller K, Ooms J, Robinson D, Seidel DP, Spinu V, Takahashi K, Vaughan D, Wilke C, Woo K, Yutani H (2019). "Welcome to the tidyverse." *Journal of Open Source Software*, *4*(43), 1686. doi:10.21105/joss.01686 https://doi.org/10.21105/joss.01686.
- Wickham H, François R, Henry L, Müller K, Vaughan D (2023). *dplyr: A Grammar of Data Manipulation*. R package version 1.1.4, https://CRAN.R-project.org/package=dplyr.

- Wickham H, Henry L (2023). *purrr: Functional Programming Tools*. R package version 1.0.2, https://CRAN.R-project.org/package=purrr.
- Wickham H, Hester J, Bryan J (2024). *readr: Read Rectangular Text Data*. R package version 2.1.5, https://CRAN.R-project.org/package=readr.
- Wickham H, Pedersen T, Seidel D (2023). *scales: Scale Functions for Visualization*. R package version 1.3.0, https://CRAN.R-project.org/package=scales.
- Wickham H, Vaughan D, Girlich M (2024). *tidyr: Tidy Messy Data*. R package version 1.3.1, https://CRAN.R-project.org/package=tidyr.
- Wilke C (2024). *cowplot: Streamlined Plot Theme and Plot Annotations for 'ggplot2'*. R package version 1.1.3, https://CRAN.R-project.org/package=cowplot.
- Xie Y (2024). *knitr: A General-Purpose Package for Dynamic Report Generation in R*. R package version 1.48, https://yihui.org/knitr/. Xie Y (2015). *Dynamic Documents with R and knitr*, 2nd edition. Chapman and Hall/CRC, Boca Raton, Florida. ISBN 978-1498716963, https://yihui.org/knitr/. Xie Y (2014). "knitr: A Comprehensive Tool for Reproducible Research in R." In Stodden V, Leisch F, Peng RD (eds.), *Implementing Reproducible Computational Research*. Chapman and Hall/CRC. ISBN 978-1466561595.
- Zhu H (2024). *kableExtra: Construct Complex Table with 'kable' and Pipe Syntax*. R package version 1.4.0, https://CRAN.R-project.org/package=kableExtra.
